# Mass & secondary structure propensity of amino acids explain their mutability and evolutionary replacements

**DOI:** 10.1038/s41598-017-08041-7

**Published:** 2017-08-10

**Authors:** Hugo J. Bohórquez, Carlos F. Suárez, Manuel E. Patarroyo

**Affiliations:** 1 Bio-mathematics, Fundación Instituto de Inmunología de Colombia, FIDIC, Cra. 50 No. 26-00, Of. 102, Bogotá DC, 111321160 Cundinamarca Colombia; 2 Universidad de Ciencias Aplicadas y Ambientales, UDCA, Bogotá DC, Colombia; 3 Universidad del Rosario, Bogotá DC, Colombia; 4 Universidad Nacional de Colombia, Bogotá DC, Colombia

## Abstract

Why is an amino acid replacement in a protein accepted during evolution? The answer given by bioinformatics relies on the frequency of change of each amino acid by another one and the propensity of each to remain unchanged. We propose that these replacement rules are recoverable from the secondary structural trends of amino acids. A distance measure between high-resolution Ramachandran distributions reveals that structurally similar residues coincide with those found in substitution matrices such as BLOSUM: Asn ↔ Asp, Phe ↔ Tyr, Lys ↔ Arg, Gln ↔ Glu, Ile ↔ Val, Met → Leu; with Ala, Cys, His, Gly, Ser, Pro, and Thr, as structurally idiosyncratic residues. We also found a high average correlation ($\overline{R}$ = 0.85) between thirty amino acid mutability scales and the *mutational inertia* (*I*_*X*_), which measures the energetic cost weighted by the number of observations at the most probable amino acid conformation. These results indicate that amino acid substitutions follow two optimally-efficient principles: (a) amino acids interchangeability privileges their secondary structural similarity, and (b) the amino acid mutability depends directly on its biosynthetic energy cost, and inversely with its frequency. These two principles are the underlying rules governing the observed amino acid substitutions.

## Introduction

In molecular evolution, protein stability is a solid indicator of function preservation thanks to a positive correlation between protein functionality and native stability^1,2.^ Natural protein sequences evolved to avoid aggregation and increase functional diversity^3^, and once a protein fold is established, the selection pressure at most positions in the protein will preserve fold stability. Homologous families of proteins have related functions, and structures are similar although sequences have diverged^4^, even in regions with less than 30% sequence identity^5,6^. Accordingly, mutation events over time may replace a residue by another while keeping the backbone dihedral angles at that position unchanged^7^. These facts indicate that the amino acid sequence alone is an incomplete measure of evolutionary relationships between proteins. Indeed, structural similarities better reflect homology than sequence similarities^8^. Therefore, sequence variation around a conserved molecular architecture could be traced through amino acid substitution patterns fixed during protein evolution.

The intrinsic secondary structure propensities of amino acids are given by the statistics of Ramachandran distributions^9–11^. In this way, we could know the conformational bias of each amino acid towards specific secondary structures^12,13^. For instance, long polypeptide chains with the same backbone conformation are found exclusively in *α* − *helix*, *PPII*, and *β* strands structures^14^. In general, examining the frequency of occurrence of particular amino acid residues in stable secondary structures have been useful for determining protein structure, folding, and energetics^15^. We propose that, in addition, the statistics of the secondary structure of proteins may reveal their evolutionary information.

**Figure 1:**
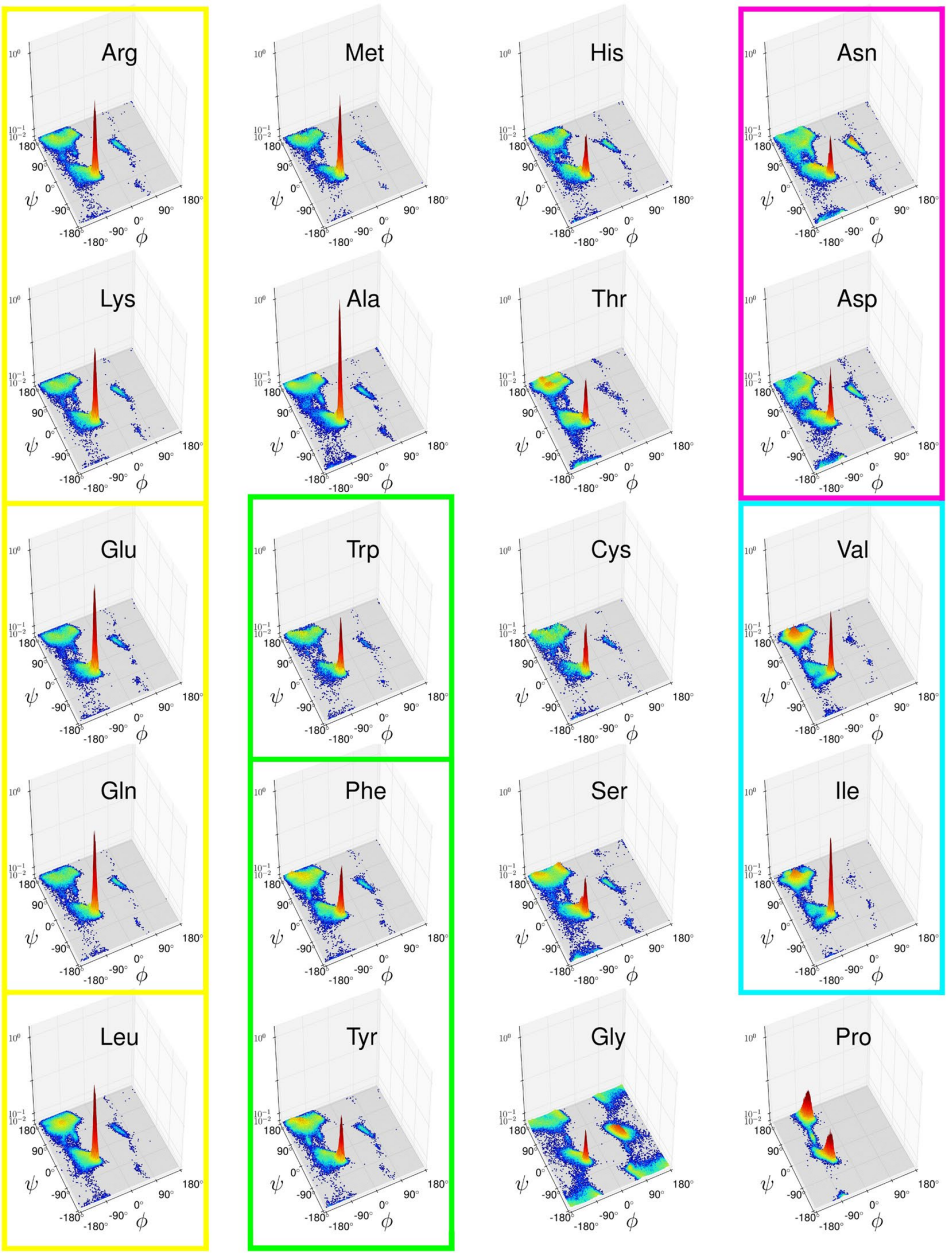
High-resolution Ramachandran probability distributions *P*_*X*_(*ϕ*, *ψ*) (logarithmic scale) as derived from the PGD 1.1 database at 1.895° × 1.895° bin size. Structurally similar open sets: yellow, *S*_*I*_ = {{Arg, Lys}, {Glu, Gln}, Leu}; green, *S*_*II*_ = {Trp, {Phe, Tyr}}; magenta, *S*_*III*_ = {Ans, Asp}; cyan, *S*_*IV*_ = {Val, Ile}. Ala, Met, and Ser have their first neighbor in *S*_*I*_; His, Thr, and Cys are adjacent to *S*_*II*_. Larger images of each Ramachandran distribution are given by Supplementary Figs. S1–S20.

**Figure 2:**
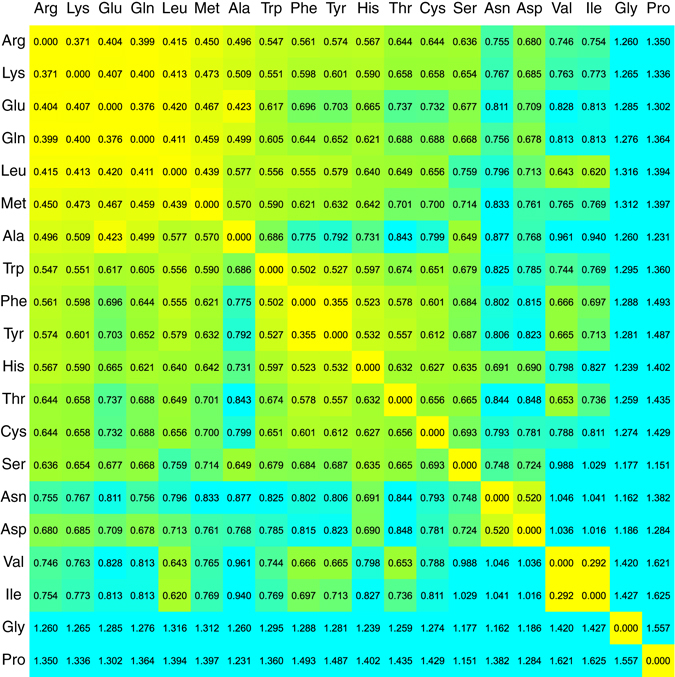
Distance matrix ordered according to structurally similar amino acids. The smallest distance is represented in yellow, and the largest distance in blue, with intermediate values in green. Open subsets appear, consistently, in yellow. Additionally, Gly, and Pro appear as the most distant elements, followed by Asn, Val-Ile, Ala, and Thr.

To confirm this assumption, we explore a combination of extensive physical quantities with the statistics of Ramachandran distributions *P*_*X*_(*ϕ*, *ψ*). In particular, we investigate the molecular mass as a measure of the amino acids biosynthetic cost. In addition, we use the protein geometry database (PGD 1.1)^16^ for obtaining high-resolution Ramachandran distributions as 2D-binned probability histograms (Fig. 1). This choice has some practical advantages, including the possibility of directly applying distance measures between the distributions. The secondary structure distance between the amino acids (Fig. 2) is the main task in our research because the emerging close-distance pairs can be straightforwardly compared to pairwise mutations. The optimal bin area (Δ*ϕ*Δ*ψ*) dividing the Ramachandran map is given by the method of Shimazaki & Shinomoto^17^. This is a key element in histogram binning because a very small bin size will result in noise amplification whereas a very large value will overpass important details of the distribution.

We explore the twenty amino acid distributions through some of their distinctive features such as the most probable conformation, which is given by the highest peak of each distribution. Additionally, we propose a plausible mutability parameter that combines structural information with the molecular mass of the amino acids. Our results indicate that amino acid evolutionary substitutions occur by following two optimal-efficiency principles: (a) interchangeability between amino acids occurs by preserving secondary structural propensity, and (b) the mutability of an amino acid depends directly on its mass, and inversely with its frequency. The methodology introduced here gives the basis for developing a new kind of scoring matrices involving physical quantities and secondary structure statistics. Hopefully, these future efforts will further help to improve the peptide design strategies, which can contribute to close the gap between the primary sequence and the 3D structure of proteins.

## Results and Discussion

### High-resolution Ramachandran Probability Distributions

We distinguish two concepts regarding the backbone dihedral angles of proteins, as suggested by Dunbrack Jr. *et al*.^11^. The first is a *Ramachandran plot* or *Ramachandran map*, which is simply a scatter plot of the *ϕ*, *ψ* values for the amino acids in a single protein structure or a set of protein structures. It provides a simple view of the conformation of a protein. The second is a *Ramachandran probability distribution P*(*ϕ*, *ψ*) which is a statistical representation of Ramachandran data, usually in the form of a probability density function. *P*_*X*_(*ϕ*, *ψ*) gives the probability of finding an amino acid conformation in a specific range of (*ϕ*, *ψ*) values.

We obtained non-parametric density estimates of *P*_*X*_(*ϕ*, *ψ*) for each amino acid *X* from 1,153,791 residues retrieved from the high-resolution protein geometry database (PGD 1.1)^16^. In our approach—frequentist—events have a specific probability whose determination depends on the number of observations. Therefore each distribution *P*_*X*_(*ϕ*, *ψ*) is given by a joint histogram. Such an approach depends on finding an optimal grid size, which can be determined with Shimazaki & Shinomoto method^17^. Said strategy requires a heuristic exhaustive sampling of a cost function whose minimum corresponds to an optimal binning of the distribution—see methods for details. Table 1 reports the optimal bin width for each Ramachandran probability distribution, ${{\rm{\Delta }}}_{X}^{min}$. The weighted average of these optimal bin widths gave us the bin size used (1.895°) in the present study. Thus, we obtained a grid with a total of 190 × 190 bins (36,100), each one covering an area of 1.895° × 1.895° of the dihedral space (Fig. 1), which is a significant improvement on the resolution of Ramachandran distributions previously reported.

**Table 1:** Properties of the Amino acids used in the present study.

Amino acid	*M*_*X*_ (Da)	*B*_*X*_	${{\boldsymbol{\Delta }}}_{{\boldsymbol{X}}}^{{\boldsymbol{\min }}}$	${{\boldsymbol{P}}}_{{\boldsymbol{X}}}^{{\bf{\max }}}$ (%)	*N*_*X*_	*W*_*X*_	*I*_*X*_
Ala	71.079	4	1.176°	0.437	113609	496.654	0.143
Arg	156.188	10	1.593°	0.265	45373	120.333	1.298
Asn	114.104	2	2.535°	0.156	46573	72.701	1.569
Asp	115.089	1	2.169°	0.192	56963	109.191	1.054
Cys	103.139	5	2.951°	0.173	15823	27.298	3.778
Gln	128.131	2	2.118°	0.307	35633	109.470	1.170
Glu	129.116	1	1.748°	0.321	48458	155.431	0.831
Gly	57.052	5	2.118°	0.124	98983	122.840	0.464
His	137.141	13	2.609°	0.173	27675	47.910	2.862
Ile	113.159	7	1.488°	0.285	74768	213.090	0.531
Leu	113.159	7	1.463°	0.276	116941	322.560	0.351
Lys	128.174	10	1.856°	0.276	40135	110.584	1.159
Met	131.193	7	1.782°	0.284	20968	59.610	2.201
Phe	147.177	11	2.169°	0.190	56511	107.242	1.372
Pro	97.117	4	2.222°	0.110	54555	60.167	1.614
Ser	87.078	4	1.978°	0.141	66612	93.593	0.930
Thr	101.105	6	2.069°	0.178	68557	121.726	0.831
Trp	186.213	14	2.687°	0.200	21118	42.340	4.398
Tyr	163.176	11	2.400°	0.184	48972	90.250	1.808
Val	99.133	4	1.622°	0.241	95564	230.082	0.431

*M*_*X*_ is the residue average mass (without water). *B*_*X*_ gives Davis’ biosynthetic steps^37^. ${{\rm{\Delta }}}_{X}^{min}(deg)$ is the optimal bin angle determined by MISE method^17^. ${P}_{X}^{{\rm{\max }}}$ corresponds to the peak of the Ramachandran distribution *P*_*X*_(*ϕ*, *ψ*). *N*_*X*_ is the number of points used for determining *P*_*X*_(*ϕ, ψ*). ${W}_{X}={P}_{X}^{{\rm{\max }}}\times {N}_{X}$ is an estimator of the maximum possible observations at the most frequent conformation. *I*_*X*_ = *M*_*X*_/*W*_*X*_ is the *mutational inertia*.

For comparison, the 3D representation of the Ramachandran distributions for the first version of PGD uses a grid of 20.0° × 20.0° (i.e. a total of 324 bins), from a dataset containing 72,376 residues^10^. In another approach, the predicted protein backbone torsion angles from NMR chemical shifts made by the TALOS+ program uses an identical bin size (20.0° × 20.0°)^18,19^, other studies on folding trends uses a resolution of 10.0° × 10.0° (i.e 1,296 bins)^11^. An early report on detailed Ramachandran distributions used bin widths of 4.0° × 4.0° (i.e. 90 × 90 bins), involving 237,384 amino acids from 1,042 proteins^20.^ Our distributions have a resolution 4.5 times higher, which translates into a higher accuracy in the distance computations between the set of distributions *P*_*X*_(*ϕ*, *ψ*). This high resolution was possible thanks to the fact that at least 84% of the structures reported at the protein data bank (PDB) were obtained during the last decade alone, most of which have atomic resolution.

Figure 1 reports the 3D plots of the twenty Ramachandran distributions determined for the present study; the dihedral angles are given in degrees, while the percentage probability per bin is given on a logarithmic scale. All the plots have the same height to facilitate their comparison. Larger plots are included in Supplementary Figs. S1–S20. While most distributions look similar one to another, there are some key differences. The probability distribution of glycine is very symmetrical and occupies all the allowed regions of the Ramachandran map. It is the only residue having a maximum at the left-handed *α*-helix conformation with a peak almost as high as the one at the *α*-helix region; these features are a consequence of its lack of a side chain^21^. On the other hand, proline—an imino acid—has two highly-populated states, with a slightly higher probability at the PPII conformation than at the *α*-helix conformation. It belongs to the set of structurally restricted amino acids composed by {Ile, Pro, Thr, Val}, which have an extremely low probability of occupying the right-hand side of the Ramachandran map. Indeed, the corresponding plots (Fig. 1) show few points within the quadrants I and IV (*ϕ* > 0). The conformational restrictions of proline arise from its pyrrolidine ring, whose flexibility is coupled to the backbone^22^. Isoleucine, threonine, and valine are the only amino acids with C-*β* branching, which means that they have more bulkiness near to the protein backbone than the rest of amino acids^23^. They also have a local maximum within the *β*-sheet region—shown as red shaded peaks in Fig. 1—a feature only shared with the three aromatic residues, Phe, Tyr, Trp, and Leu. The remaining amino acids occupy the allowed regions in a generic fashion^20,24^, whose distributions agree with the original Ramachandran and co-workers explanation in terms of steric clashes^25^.

All these observations point to the qualitative aspects of the distributions. However, a systematic comparison of the twenty Ramachandran distributions requires the use of a quantitative evaluation of their similarities. In the following subsection, we show a distance matrix accounting for dissimilarities between the secondary-structural trends of amino acids.

### Secondary-structural vs BLOSUM replacements

A quantitative assessment of the similarities between the twenty distributions *P*_*X*_(*ϕ*, *ψ*) requires a distance measure. We used the *city-block* distance, which can be used to assess the differences in discrete frequency distributions. It gives more weight to the most probable dihedral conformations of the Ramachandran distributions.

Each amino acids *X* has a set of twenty distances, *D*_*X*_, including with itself, (in which case ||*P*_*X*_ − *P*_*X*_|| = 0):

1 $${D}_{X}=\{||{P}_{X}-{P}_{Ala}||,||{P}_{X}-{P}_{Arg}||,\ldots ,||{P}_{X}-{P}_{Tyr}||,||{P}_{X}-{P}_{Val}||\}$$

The most plausible secondary-structural replacement to *X* is that amino acid *Y* having the smallest positive distance to *X*, or the minimum positive value from the set of distances: ${min}_{+}\{{D}_{X}\}$. That ${{\rm{\min }}}_{+}\{{D}_{X}\}=||{P}_{X}-{P}_{Y}||$ does not imply necessarily that ${{\rm{\min }}}_{+}\{{D}_{Y}\}=||{P}_{Y}-{P}_{X}||$. In other words, the structural replacement is not always a reciprocal operation; hence if *Y* is the replacement of *X*, we denote this by *X* → *Y*. In the case of a reciprocal replacement, we denote it by *X* ↔ *Y*.

The secondary-structural distance matrix between the amino acids is shown in Fig. 2. The proximity between amino acids is given by a color scheme: the smallest distance is represented in yellow, and the largest distance in blue, with intermediate values in green. We found *open subsets* by a nearest-neighbor criterion: any element within an open subset has exactly the remaining elements of said subset as its nearest neighbors—the procedure is explained in the methods section. For instance, the simplest open subset is composed by two elements for which the other one is the closest element—i.e. those elements for which *D*_*min*_(*P*_*X*_, *P*_*Y*_) = *D*_*min*_(*P*_*Y*_, *P*_*X*_) or, equivalently, *X* ↔ *Y*.

**Figure 3:**
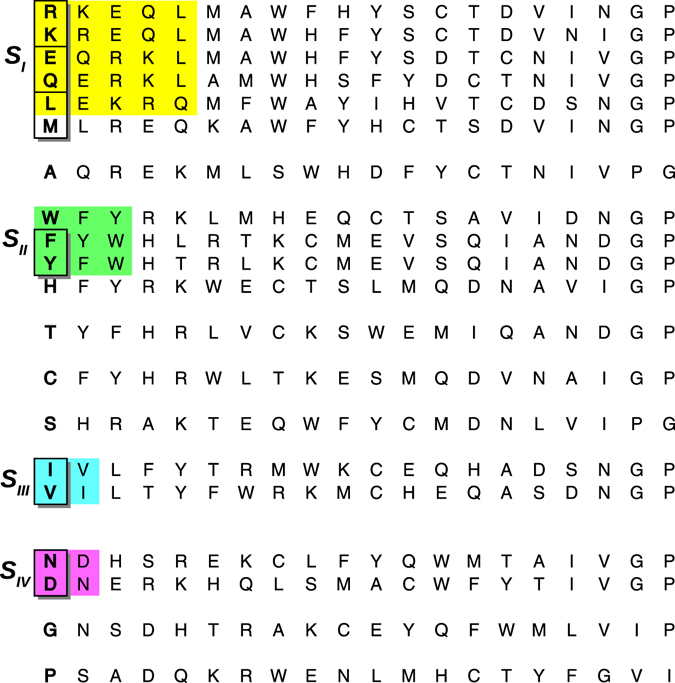
Rows ordered according to the cityblock distance. Open sets are indicated by the same color code used in Fig. 1. The shadowed boxes contain the BLOSUM100 pair replacements. The procedure for determining an open set consists on finding rows with the same set of first neighbors. For instance, the first neighbor of Arg (top row) is Lys; after placing the Lys row under the top row, we see that they share the seven first neighbors (up to Trp). The third row corresponds to Arg second neighbor, i.e. Glu, which also shares the same first neighbors with the previous ones up to Trp. The fourth row corresponds to Arg third neighbor, i.e. Gln, whose fifth neighbour is Ala, unlike the previous rows. The fifth row corresponds to Arg fourth neighbor, i.e. Leu, which has all the previous rows as its first neighbors. In this way, the yellow box includes those elements whose first four neighbors are completely contained within the set. Methionine is a frontier element of this set: its first five neighbors are exactly the elements of the whole closed set; however, Glu does not include Met within its first five neighbours and for that reason Met is not contained in the set. The remaining open sets *S*_*II*_ to *S*_*IV*_ were obtained in the same way. Notice that Pro and Gly are the farthest residues from any other one, as a consequence of their structural propensity uniqueness.

We found the following open sets (Fig. 3): a five-member set including a couple of two-member subsets: *S*_*I*_ = {{Arg, Lys}, {Glu, Gln}, Leu}—in yellow; a three-member set containing a two-member set, *S*_*II*_ = {Trp, {Phe, Tyr}}—in green; and a pair of two-member sets: *S*_*III*_ = {Val, Ile}, and *S*_*IV*_ = {Asn, Asp}—in cyan and magenta, respectively. Within this topology, Met appears as a boundary element of the first set *S*_*I*_; Fig. 3 shows that Met first five neighbors are exactly the elements of *S*_*I*_. In turn, every residue in *S*_*I*_ has Met as the fifth neighbor but Glu, which has Ala closer; this proximity may result from Ala and Glu being the strongest *α*-helix formers, as their respective ${P}_{X}^{{\rm{\max }}}$ values indicate (Table 1). The *S*_*I*_ group includes aliphatic saturated side chains, while *S*_*II*_ contains the aromatic residues. Adjacent to these two major sets we found residues sharing their physiochemical characteristics—as shown by their close distances to the main groups in the distance matrix (Fig. 2). Specifically, four residues have their nearest neighbor within a major open set: Ala have its first neighbor in *S*_*I*_, whereas His, Thr, and Cys have their first neighbor in *S*_*II*_. Those amino acids outside an open set or its boundaries were considered structurally idiosyncratic: Ala, Cys, His, Gly, Ser, Pro, and Thr. Gly and Pro are the farthest ones from any other residue, as the last column of Fig. 3 shows. Certainly, these amino acids populate the Ramachandran map in a unique way. The Ramachandran distribution of glycine is widespread over the allowed regions; while Pro is the most structurally restricted. Alanine has twice the probability of forming an *α*-helix (${P}_{Ala}^{{\rm{\max }}}=\mathrm{0.437 \% }$ from Table 1) than any other residue (${P}_{aver\ne Ala}^{{\rm{\max }}}=0.214 \%$). The Ramachandran distribution of Thr has four peaks around the *β* and *π* regions unlike any other residue, including the C-*β* branched amino acids (Fig. 1). While Thr is chemically similar to Ser^26^, they have different structural propensities. According to our distance matrix (Fig. 2), Thr is closer to Tyr & Phe, while Ser is closer to His & Arg. A recent study shows that the phosphorylation of Ser increases its propensity of forming PPII, whereas that of Thr has the opposite effect^27^. This result indicates that Ser and Thr are far from being ideal secondary structural replacements. In summary, our classification reflects the intrinsic structural trends of amino acids; in particular, the *S*_*I*_ set and its adjacent elements Met and Ala are the same alpha formers found by Fujiwara *et. al*.^28^. Within the same scale, the aromatic set, *S*_*II*_, and its adjacent elements (Cis, Thr) and *S*_*III*_ are beta formers. The remaining amino acids are turn/bend formers, including *S*_*IV*_ and Gly, Ser, and Pro, most of which have the lowest ${P}_{X}^{max}$ values in Table 1.

More importantly, nevertheless, is the fact that an unexpected pattern emerged: our structurally similar pairs of amino acids matches with most BLOSUM matrices pair replacements^29^, which are shown as shadowed boxes in Fig. 3. More details about the substitution matrices are in the methods section. Our list of structural replacements is: Asn ↔ Asp, Phe ↔ Tyr, Lys ↔ Arg, Gln ↔ Glu, Ile ↔ Val, Met → Leu. In BLOSUM matrices, Thr and Ser are replacements. For all BLOSUM matrices, Gly, Pro, Cys, His, and Ala are idiosyncratic residues. In general, our set of structurally-similar amino acids coincide with most canonical residue substitutions given by scoring matrices such as BLOSUM62 and BLOSUM100^29^, and consensus replacements^30^. This is a remarkable finding considering the extremely low probability of randomly finding six out of seven replacement pairs: less than one in a 681 million, as detailed in the methods section. In consequence, our result reveals an underlying correlation between mutation matrices and structural propensities. Hence, the replacement rules implied by the secondary structure distance (Fig. 2) may be directly used for for exploring structural amino acid replacements in peptide design strategies.

We conclude that during evolution, mutational replacements occurred between structurally similar amino acids. Hence, mutations followed a process that privileges structure and hence preserves function. But BLOSUM and PAM substitution matrices give additional information about the mutational trends of amino acids. The diagonal of these matrices determine how easy is for an amino acid to be replaced. A large value means more resistance to change. However, our distance matrix (Fig. 2) has a diagonal of zeros. For studying the mutability, we explored a parameter that combines the statistical information at the ${P}_{X}^{{\rm{\max }}}$ with a basic extensive property.

### Molecular mass and optimum evolutionary cost

Molecular mass is a fundamental extensive property that might have played a central role in defining the actual protein landscape. Previously, our group revealed a very high correlation (*R* = 0.98) between mass and the electronic energy of amino acids—excluding the two sulfur-containing side chains^31^. In the present study, we found a complex relationship between the amino acids mass *M*_*X*_ and the structural trends via the probability at ﻿the most frequent conformational state,  ${P}_{X}^{{\rm{\max }}}$; this quantity is given by the highest peak of each Ramachandran distribution—max(*P*_*X*_(*ϕ*, *ψ*)). ${P}_{X}^{{\rm{\max }}}$ corresponds to the most frequent conformation and, therefore, it is an indicator of structural persistence^32^.

The *α*-helix conformation is the highest peak for all amino acids (but proline) with alanine at the top as the strongest helix former. While mass has an overall poor correlation with ${P}_{X}^{{\rm{\max }}}$ (R = 0.05), we identified two main and opposite trends delimited by separate ranges of ${P}_{X}^{{\rm{\max }}}$: (a) ${P}_{X}^{{\rm{\max }}} > \mathrm{0.200 \% }$ defines the set of strong helix formers {Ala, Glu, Gln, Ile, Met, Leu, Lys, Arg, Val} (in descending order), with a negative correlation *R* = −0.61; and, (b) ${P}_{X}^{{\rm{\max }}}\le \mathrm{0.200 \% }$ defines the weak helix formers: {Trp, Asp, Phe, Tyr, Thr, His, Cys, Asn, Ser, Gly, Pro}, with a positive correlation of *R* = 0.76. The small set of C-*β* branched amino acids ({Ile, Thr, Val}) plus proline shows a correlation of *R* = 0.78 between mass and ${P}_{X}^{{\rm{\max }}}$. After excluding these four elements from the two main sets, their respective correlations rise to *R* = −0.87 for the strong helix formers, and to *R* = 0.87 for the set of weak helix formers. In strong helix formers, the negative correlation between ${P}_{X}^{{\rm{\max }}}$ and the molecular mass indicates that light side chains have a better chance of forming an alpha helix than heavy ones. These three correlations reveal a direct involvement of the molecular mass on the *α*-helical propensities of the amino acids.

**Figure 4:**
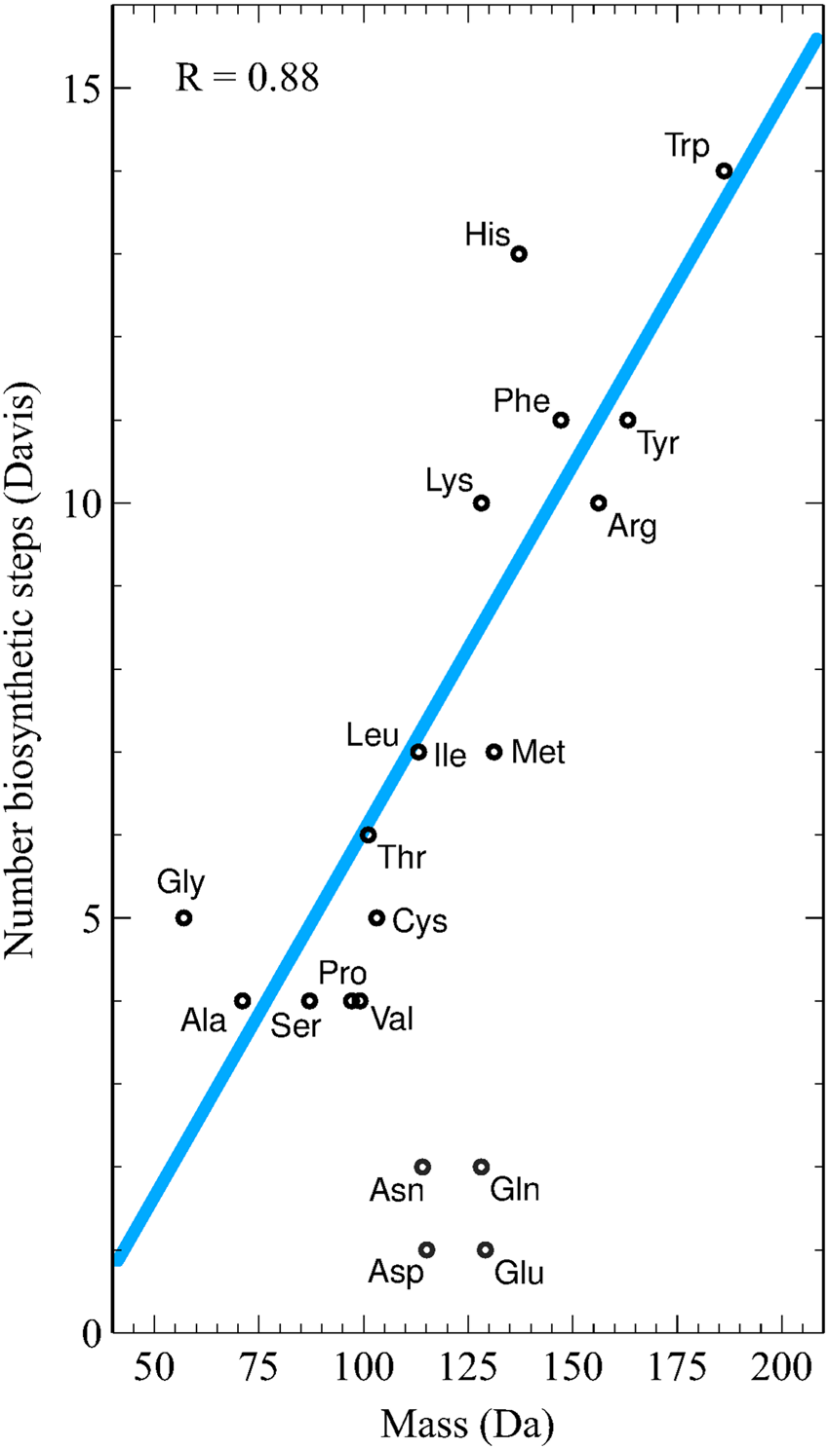
Correlation between the molecular mass of the amino acids *M*_*X*_ and their energetic cost as accounted by the number of biosynthetic steps *B*_*X*_ proposed by Davis^37^. The outliers {Asn, Asp, Gln, Glu} are excluded from the Pearson’s correlation and from the linear interpolation.

A recent observation by Lehmann *et. al*. reports a negative correlation between the background frequency and codon degeneracy of amino acids with mass^33^. Seligmann already observed that the evolutionary rate of amino acid replacements correlates negatively with mass^34^. Accordingly, heavier amino acids are less frequent, which suggests that the genomes preserve a fundamental distribution ruled by simple energetics. Inverse correlations between the average amino acid biosynthetic cost and the levels of gene expression are consistent with natural selection to minimize costs^35^. Seligmann also shows a positive correlation (*R* = 0.80) between the molecular mass *M*_*X*_ and the total energetic cost per amino acid (in ATPs)^34^, as reported by Akashi & Gojobori^36^. According to Lehmann *et al*., highly expressed proteins tend to use amino acids with relatively low synthetic costs^33^. Therefore, heavy amino acids are less frequent because they are biosynthetically more expensive. We found a further confirmation of this statement: the molecular mass grows with the number of biosynthetic steps, as shown in Fig. 4. The values proposed by Davis^37^, are included in Table 1 as *B*_*X*_. The number of biosynthetic steps has been proposed as a natural way of determining the evolutionary history of amino acids^38^, and so does the amino acids molecular mass. We found a correlation of *R* = 0.64 between mass and biosynthetic steps, which rises up to *R* = 0.88 after excluding the set of outliers {Asn, Asp, Gln, Glu} (Fig. 4).

In summary, we found a high correlation—by parts—between the molecular mass and the probability at the most frequent conformational state (${P}_{X}^{{\rm{\max }}}$). We also found a high correlation between mass and the number of biosynthetic steps (*B*_*X*_). These correlations are consistent with the fact that evolution privileges energetically optimal costs^34,39^. Thus, in the quest for a physical quantity that can explain amino acid’s mutability, mass is irreplaceable as a fundamental measure of energetic cost.

### Mass over the frequency at the most probable conformation correlates with mutability

The background frequency or natural abundance of amino acids, *N*_*X*_, may be indicative of their evolutionary age: more abundance reflects an early adoption in molecular evolution^40^. The values of *N*_*X*_ were obtained from the PGD 1.1 database (Table 1). The quantity ${W}_{X}={P}_{X}^{{\rm{\max }}}\times {N}_{X}$ is an estimator of the maximum observations at the most frequent conformation. In this way, *W*_*X*_ combines the probability at the most probable conformation with the background frequency. In the previous section we showed that an amino acid has less probability to be changed if it is more energetically expensive, and therefore mass directly measures the resistance to be changed. Additionally, less frequent amino acids are also less replaceable, indicating an inverse correlation with the mutability. Under these considerations, we define a “replacement inertia” as the mass *M*_*X*_ weighted by *W*_*X*_: *I*_*X*_ = *M*_*X*_/*W*_*X*_. It summarizes the energetic cost per number of observations at the most probable conformation. We hypothesize that *I*_*X*_ might reflect the mutability of amino acids—i.e. the diagonal of substitution matrices (see more details in the Methods).

**Figure 5:**
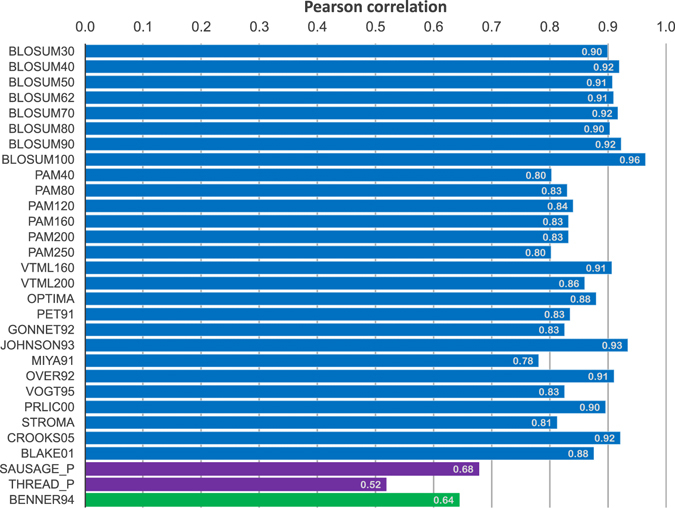
Pearson correlation coefficients between the replacement inertia *I*_*X*_ (Table 1) and the mutability of thirty replacement matrices. Alignment derived matrices are shown in blue, force field derived matrices in purple, and the genetic code derived matrix in green. See Supplementary Table S1 for the abbreviations.

In order to test if *I*_*X*_ reflects the mutability of amino acids, we selected thirty replacement matrices reported by the AAindex^41^: twenty-seven that were built from sequence alignments—including a selection of six PAM and eight BLOSUM matrices; two more that were crafted from force fields (THREADER and SAUSAGE)^42^; and a last one that was obtained from replacements at the genetic code level^43^. Supplementary Table S1 contains the list of matrices used in our survey. We computed the Pearson correlation coefficient between *I*_*X*_ and each mutability, which is shown in Fig. 5; in this figure, the correlation with alignment-derived matrices is colored in blue; the correlation with force-field derived appears in purple; and the correlation with the genetic code based matrix is plotted in green.

We found a very strong average correlation between *I*_*X*_ and the whole mutability set of ${\overline{R}}_{30}=0.85$. This average value can be explained by the strong correlation found between *I*_*X*_ and the mutability of matrices derived from sequence alignments, which have values *R* > 0.78, as Fig. 5 shows. For the family of BLOSUM matrices, R values were obtained between 0.90 and 0.96, with an average correlation of ${\overline{R}}_{B}=0.92$. For PAM matrices, the correlation was lower with an average value of ${\overline{R}}_{P}=0.82$ for the six PAM matrices included in our survey.

On the other hand, the correlation between *I*_*X*_ and the mutability of the THREADER substitution matrix was the lowest we found, *R*_*THREADER*_ = 0.52. The second lowest correlation for was with the matrix based on the genetic code (*R*_*BENNER*_ = 0.64). The other force field derived matrix gave a correlation of *R*_*SAUSAGE*_ = 0.68. These low correlations may have an interesting explanation: while force field based substitution matrices do not include evolutionary information, BENNER matrix, on the other hand, assumes that the genetic code is the only determinant of amino acid substitutions. As a consequence, the underlying factors controlling these matrices are poorly reflected on *I*_*X*_. Therefore, we must conclude that the very high correlation between *I*_*X*_ and the mutability of matrices derived from sequence alignments implies that molecular mass, abundance, and the most probable secondary structure conformation may have played a decisive role on shaping the molecular evolution of proteins.

**Figure 6:**
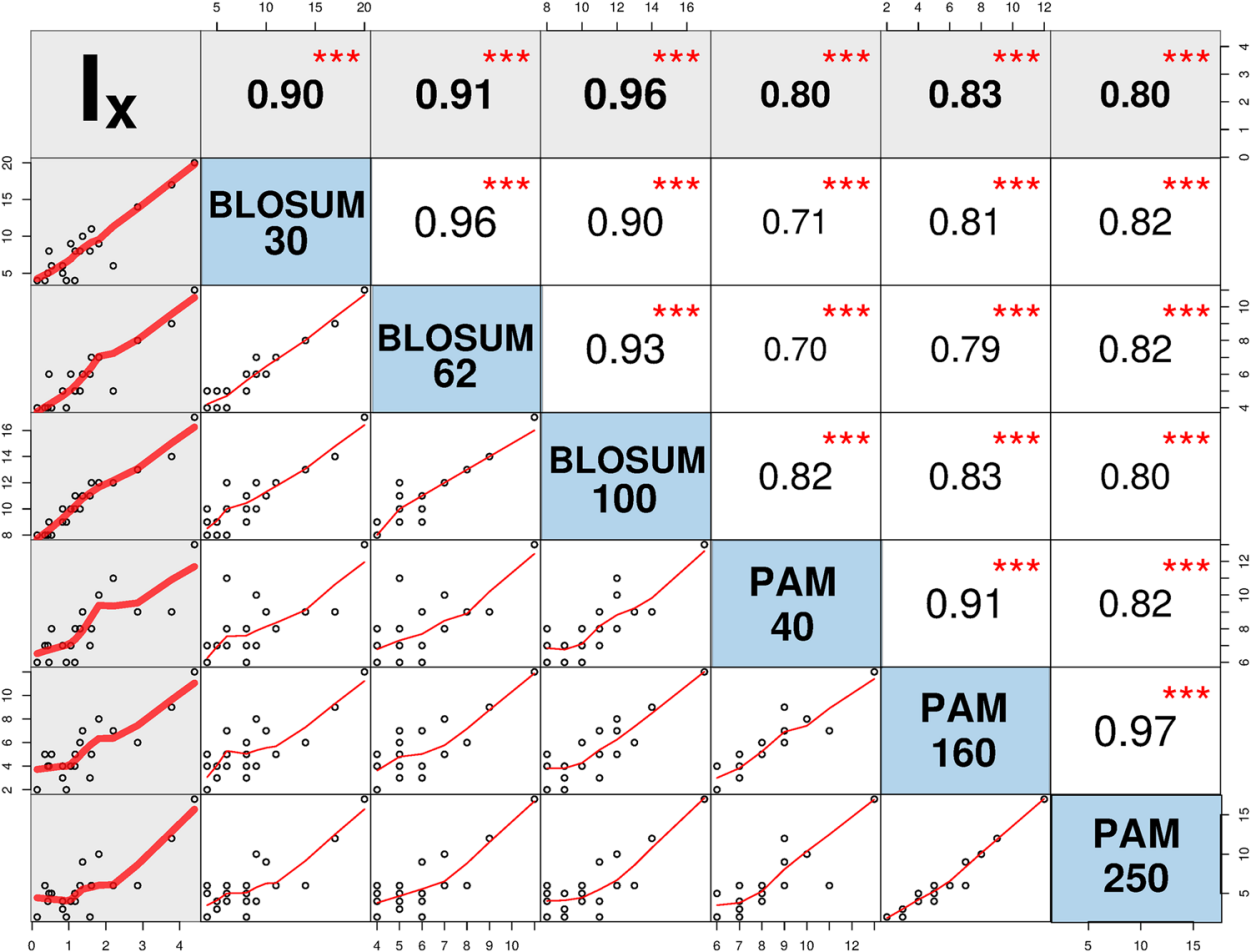
Correlation matrix plot with significance levels between the replacement inertia (*I*_*X*_) and the mutability of a representative set of BLOSUM and PAM matrices. The lower triangular matrix is composed by the bivariate scatter plots with a fitted smooth line. The upper triangular matrix shows the Pearson correlation plus significance level (as stars). Each significance level is associated to a symbol: p-values 0.001 (***), 0.01 (**), 0.05 (*). This plot was generated with the Performance Analytics package in R program^57^. The correlation matrix for the complete mutability set is plotted in Supplementary Fig. S1.

However, how significant an average correlation of $\overline{R}=0.85$ between *I*_*X*_ and the mutability set is? We evaluated the correlation coefficients between the mutability of all the substitution matrices, which yields a total of 430 correlations for the thirty matrices considered. The average value for these correlations is ${\overline{R}}_{430}=0.84$. This value differs little from $\overline{R}$, which means that *I*_*X*_ describes amino acids mutability as well as any the mutability of the accepted mutation matrices. The correlation matrix with significance levels for *I*_*X*_ and the mutability of the whole set of matrices is shown in Supplementary Fig. S1. An excerpt of this plot is shown in Fig. 6, which includes the following matrices: BLOSUM30, BLOSUM62, BLOSUM100, PAM40, PAM160, and PAM250. This plot reveals that the correlations between PAM and BLOSUM fall within 0.70 and 0.83. Expectedly, correlations between matrices of the same family are higher, up to 0.96 for BLOSUM and up to 0.97 for PAM. It is surprising that *I*_*X*_ had better simultaneous correlations with both matrix families than they have with each other. This observation holds for the eight BLOSUM and six PAM matrices included in our study, as shown in Supplementary Fig. S21.

Our results indicate that amino acids mutability may be an evolutionary invariant that depends on the biosynthetic cost per amino acid and on the background frequency. These observations might have relevant consequences for future developments and improvements of the actual scoring matrices, as well on structure prediction and design.

## Conclusions

Our study provides compelling evidence about the physiochemical nature of the substitution matrices. Taylor’s early work^44^ on *evolutionary biochemistry*^45^ proposes an integrative amino acid classification schema based on Dayhoff’s PAM matrix and properties such as volume and polarity. In a complementary way, our approach puts the evolutionary concepts closer to physiochemical properties, which might be helpful for treating proteins as integrated physical and historical wholes.

The main findings of the present work agree with accepted ideas about the molecular evolution of proteins. In the first place, we claim that secondary structural similarities resemble to a great extent the canonical replacements given by substitution matrices (Figs 2 and 3). We interpret this result as a manifestation of an underlying structural preservation principle according to which amino acids interchangeability is highly determined by their secondary structural similarity. It might be a consequence of the fact that less structurally important parts of a protein evolve faster than more important ones. In this way, conservative substitutions occur more frequently in evolution than more disruptive ones. Our result agrees with Koonin & Wolf view according to which the primary causes of protein evolution could have more to do with fundamental principles of protein folding than with unique biological functions^46^. In the second place, we showed that amino acids mutability is correlated with the *replacement inertia I*_*X*_ (Fig. 5). Therefore, amino acids mutability depends on the biosynthetic cost, the most probable conformation, and the background frequency. Davis proposes that the timeline of genetically encoded amino acids correlates with the number of chemical reactions required to synthesize each amino acid^37,38,47^. As a consequence, the correlation between mass and biosynthetic steps (Fig. 4) indicates that the mutability of amino acids might be a timeline of protein evolution as well.

Undeniably, the biosynthetic cost, structural preservation, and frequency distribution of amino acids, all played a significant role in the molecular evolution of proteins. Indeed, two main selective factors determining the evolution of proteins are structural robustness against misfolding, and energy-cost efficiency^46,48,49^. Protein synthesis is very error-prone in comparison to DNA replication, and hence many folding-recognition mechanisms seem to have evolved to minimize costs of erroneous protein synthesis^49^. This energy-cost efficiency may explain why highly expressed proteins evolve slowly and at rates largely unrelated to their functions^48^.

We can summarize our two main findings in similar terms with the following optimal-efficiency principles: (a) amino acids interchangeability occurs by preserving the secondary structural propensity, and (b) the amino acid mutability depends directly on its biosynthetic energy cost, and inversely with its frequency at the most probable conformation. We believe that these two principles are the underlying rules governing the observed amino acid substitutions. They provide a unified interpretation to mutation matrices, outside the statistical realm alone. Our results also indicate that amino acids mutability might be an invariant scale that differs little from one substitution matrix to another (Supplementary Fig. S21). These results may offer a new understanding of the evolutionary processes determining the structure of proteins.

Finally, the statistical similarities between secondary structural propensities used here offer a viable methodology for systematically exploring amino acid structural replacements. For instance, one can determine a structural distance matrix limited to the *β*-strand region, which may differ from the one of the whole Ramachandran map. With this type of sectoral statistics one can envision new rules for the design of polypeptide chains.

## Methods

### Data source

We calculated the Ramachandran distributions from the protein geometry database PGD 1.1, retrieved in June 2016^16^. We selected crystallized protein geometries with resolution equal or less than 2Å, a R-factor equals to 0.2, and a R-free maximum of 0.3. In order to avoid over-representation bias of some protein families, we used 7,398 proteins with a maximum identity of 25%. A total of 1,153,791 residues were considered.

### Data analysis

The statistical analysis of the present work was implemented in Python 2.7 programming language^50,51^. A Python routine extracts the observed (*ϕ*, *ψ*) values from the PGD database for each amino acid (PGDread.py). The 2D optimization process was done with a routine that computes the cost function by changing the bin width equally for both dihedral variables Δ = Δ*ϕ* = Δ*ψ*, (MISE.py). The Ramachandran distribution histograms were computed and plotted with Matplotlib libraries (3DRamadistr.py)^52^. The cityblock distance was taken from the SCIPY package. A total of 600 code lines were written for the complete analysis shown here. The Python codes are available upon request.

### Histogram optimization

Histograms are a type of non-parametric density estimates for which the number of parameters equals the number of data points^53^. A different approach uses analytic functions for obtaining smooth distributions that minimize low resolution and outliers effects^54^. The discrete (histogram) representation of the joint probability distribution *P*_*X*_(*ϕ*_*i*_, *ψ*_*j*_) depends on the bin width of the dihedral variables, i.e. Δ*ϕ* and Δ*ψ*. A coarse binning size decreases the data noise but it might overpass relevant details of the structural information. On the other hand, a very fine grain bin size might highlight underlying statistical noise. The mean integrated squared error (MISE) can be estimated from the data through a cost function *C*(Δ). A histogram with the bin size that minimizes the MISE is optimal^17^. This method guarantees that a substantial increasing in the observations will further increase the accuracy of the histogram representation of probability distributions even more. The main assumption underlying this method is that the distribution can be represented by a smooth continuum function. Previous works have proven that Ramachandran distributions obey such assumption^11^. We assumed a regular partitioning of the Ramachandran maps i.e having the same bin size Δ for both dihedral variables: Δ = Δ*ϕ* = Δ*ψ*. The cost function for two variables is therefore given by

2 $$C({\rm{\Delta }})=\frac{2n-v}{{{\rm{\Delta }}}^{4}}$$

where the mean *n* and the variance *v* of the number of occurrences are given, respectively, by $n=\frac{1}{N}{\sum }_{i}^{N}{n}_{i}$, and $v=\frac{1}{N}{\sum }_{i}^{N}{({n}_{i}-n)}^{2}$. The obtained optimal bin value for each amino acid is Δ_*X*_ (Table 1). We used the weighted average as the bin with for all the Ramachandran distributions: $\overline{{\rm{\Delta }}}={\sum }_{X}^{20}{N}_{X}{{\rm{\Delta }}}_{X}/{\sum }_{X}^{20}{N}_{X}$. From the obtained Δ_*X*_ values, $\overline{{\rm{\Delta }}}={1.887}^{^\circ }$, which was approximated by the integer fraction 360°/190 ≃ 1.895°, i.e. we used 190 bins in each angular coordinate, for a total of 190 × 190 = 36,100.

### Amino acid classification

We classified the amino acids according to the city-block (Manhattan) distance. Our grouping method takes advantage of the fact that a metric induces a topology on a set. Accordingly, we determined the topology induced by the city-block distance over the set of amino acids. The increasing distance between a given element *X* and the remaining ones determines an ordered list. Therefore, for the present case, we have twenty ordered lists, one for each amino acid. The intersection between the first neighbors of these lists gave us *open subsets*. An open subset consists on those elements such that, for every element within the subset, its neighbors belong to the same subset. Figure 3 reports the twenty ordered lists with an example about how to obtain open sets.

### Substitution matrices and mutability

The most common method of evaluating the amino acid substitution patterns is through substitution matrices such as PAM^55^ or BLOSUM^29^. A typical substitution matrix has 20 × 20 elements, in which non-diagonal pairwise scores (log odds) represent the probability of one amino acid could be substituted by other in protein evolution. The diagonal scores of the matrix are estimators of amino acid mutability. For each amino acid, a greater score implies lesser possibilities to be substituted, on the other hand, lesser scores implies a greater chance to be substituted^55,56^. We used a set of thirty substitution matrices reported in the AAindex^41^ and NCBI (ftp://ftp.ncbi.nih.gov/blast/matrices/).

### Probability of randomly finding six out of seven sets

Substitution matrices, such as BLOSUM62 & BLOSUM100, define seven replacement pairs of amino acids. Our structural similar pairs do coincide with six of them. We need an assessment of the probability for correctly obtaining six out of seven pairs. The probability of obtaining the first element of a pair is the number of elements of such pair (2) divided by the total of elements (14). Then, the probability of finding the match is the number of pair elements still in the set (1) divided by the total left (13). Hence, the combined probability of randomly finding the first pair out of seven is *P*_1_ = 2/14 × 1/13. By a similar reasoning, the probability of obtaining a second pair is *P*_2_ = 2/12 × 1/11, and so on. Therefore, the probability of simultaneously finding six out of seven pairs is ${\prod }_{i\mathrm{=1}}^{6}{P}_{i}$, or equivalently, ${\prod }_{k\mathrm{=2}}^{7}\frac{2}{2k(2k-1)}=\mathrm{1/681,080,400}=1.468\times {10}^{-9}$. In other words, there is a chance of one in 681 million of simultaneously obtaining six correct pairs from a set of seven pairs.

## Electronic supplementary material

Supplementary
