# Publisher Correction: Mass & secondary structure propensity of amino acids explain their mutability and evolutionary replacements

**DOI:** 10.1038/s41598-018-21981-y

**Published:** 2018-03-06

**Authors:** Hugo J. Bohórquez, Carlos F. Suárez, Manuel E. Patarroyo

**Affiliations:** 1 Bio-mathematics, Fundación Instituto de Inmunología de Colombia, FIDIC, Cra. 50 No. 26-00, Of. 102, Bogotá DC, 111321160 Cundinamarca Colombia; 2 Universidad de Ciencias Aplicadas y Ambientales, UDCA, Bogotá DC, Colombia; 3 Universidad del Rosario, Bogotá DC, Colombia; 4 Universidad Nacional de Colombia, Bogotá DC, Colombia

Correction to: *Scientific Reports* 10.1038/s41598-017-08041-7, published online 10 August 2017

The original version of this Article contained a typographical error in the Abstract.

“Asn ↔ Asp, P™he ↔ Tyr, Lys ↔ Arg, Gln ↔ Glu, Ile ↔ Val, Met → Leu”

now reads:

“Asn ↔ Asp, Phe ↔ Tyr, Lys ↔ Arg, Gln ↔ Glu, Ile ↔ Val, Met → Leu”

Additionally, there was an error in the Results and Discussion section, under the subheading ‘Mass over the frequency at the most probable conformation correlates with mutability’.

“the correlation with force-field derived appears in green; and the correlation with the genetic code based matrix is plotted in purple.”

now reads:

“the correlation with force-field derived appears in purple; and the correlation with the genetic code based matrix is plotted in green.”

These errors have now been corrected in the HTML and PDF versions of the Article.

