# Supplementary material for: Mass & secondary structure propensity of amino acids explain their mutability and evolutionary replacements
Source: Sci Rep. 2017 Aug 10;7:7717. doi: 10.1038/s41598-017-08041-7 (PMC5552740; doi:10.1038/s41598-017-08041-7)
Supplement: Supplementary file 1 — Supplementary [file 41598_2017_8041_MOESM1_ESM.pdf]

Supplementary material
Mass & secondary structure propensity of amino acids explain their mutability and evolutionary replacements

Hugo J. Bohórquez[1,+,*], **Carlos F. Suárez**[1,2,3,+], **and Manuel E. Patarroyo**[1,4]

[1]Fundación Instituto de Inmunología de Colombia, FIDIC, Biomathematics, Cra. 50 No. 26-00, Bogotá D. C., Colombia
[2]Universidad de Ciencias Aplicadas y Ambientales, UDCA, Bogotá D. C., Colombia
[3]Universidad del Rosario, Bogotá D. C., Colombia
[4]Universidad Nacional de Colombia, Bogotá D. C., Colombia
[*]hugo.j.bohorquez@fidic.org.co

ABSTRACT

We use the protein geometry database (PGD 1.1)[1] for obtaining the high-resolution Ramachandran distributions as 2D-binned probability histograms (Figures S1 to S20). The optimal bin area ($1.895^\circ \times 1.895^\circ$) dividing the Ramachandran map was obtained with the method of Shimazaki & Shinomoto.[2] Figure S21 shows the correlation matrix plot with significance levels between the replacement inertia I_X and the mutability of the full set of replacement matrices used in the present study (Table S1).

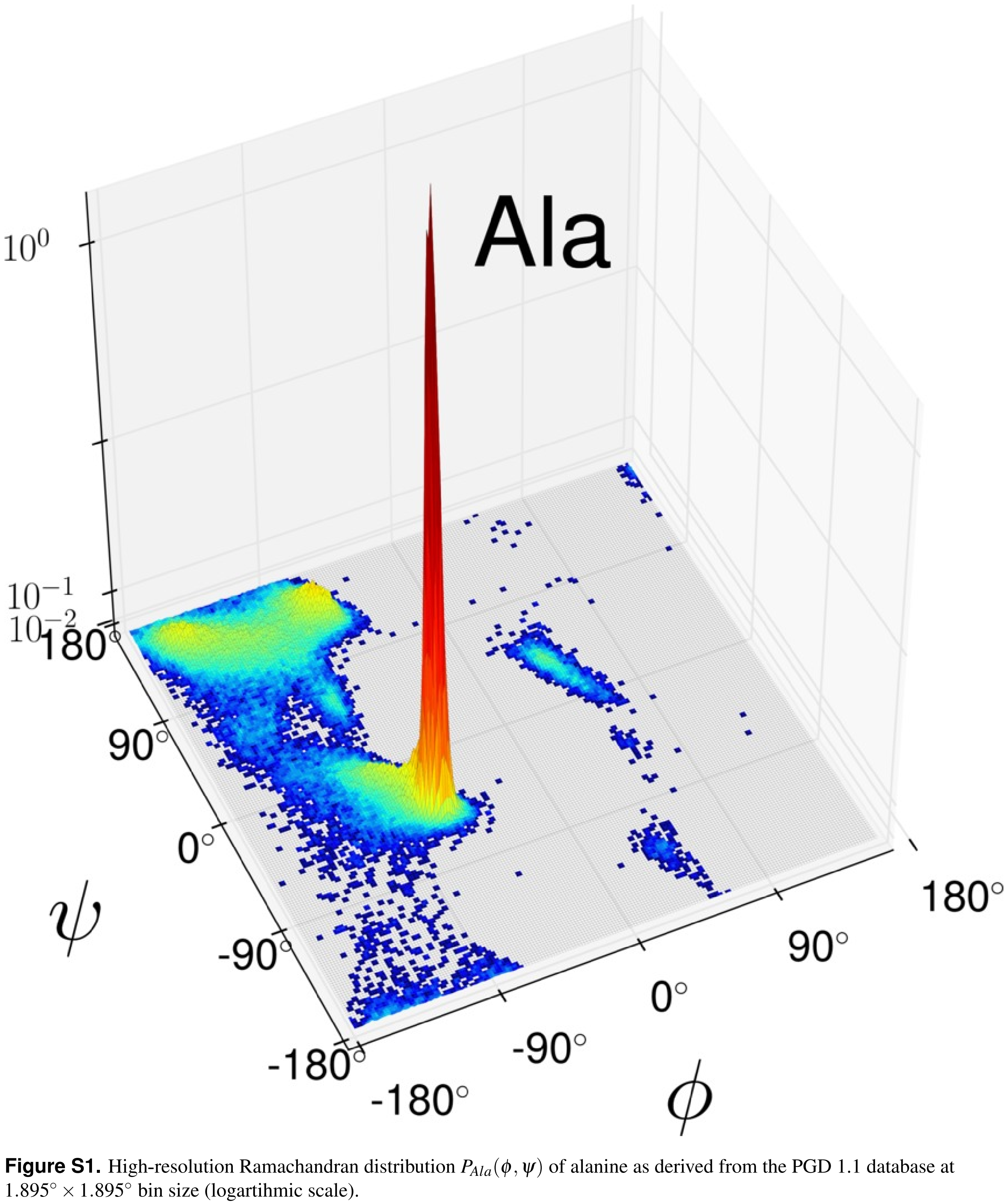

Figure S1. High-resolution Ramachandran distribution $P_{Ala}(\phi, \psi)$ of alanine as derived from the PGD 1.1 database at $1.895^\circ \times 1.895^\circ$ bin size (logartihmic scale).

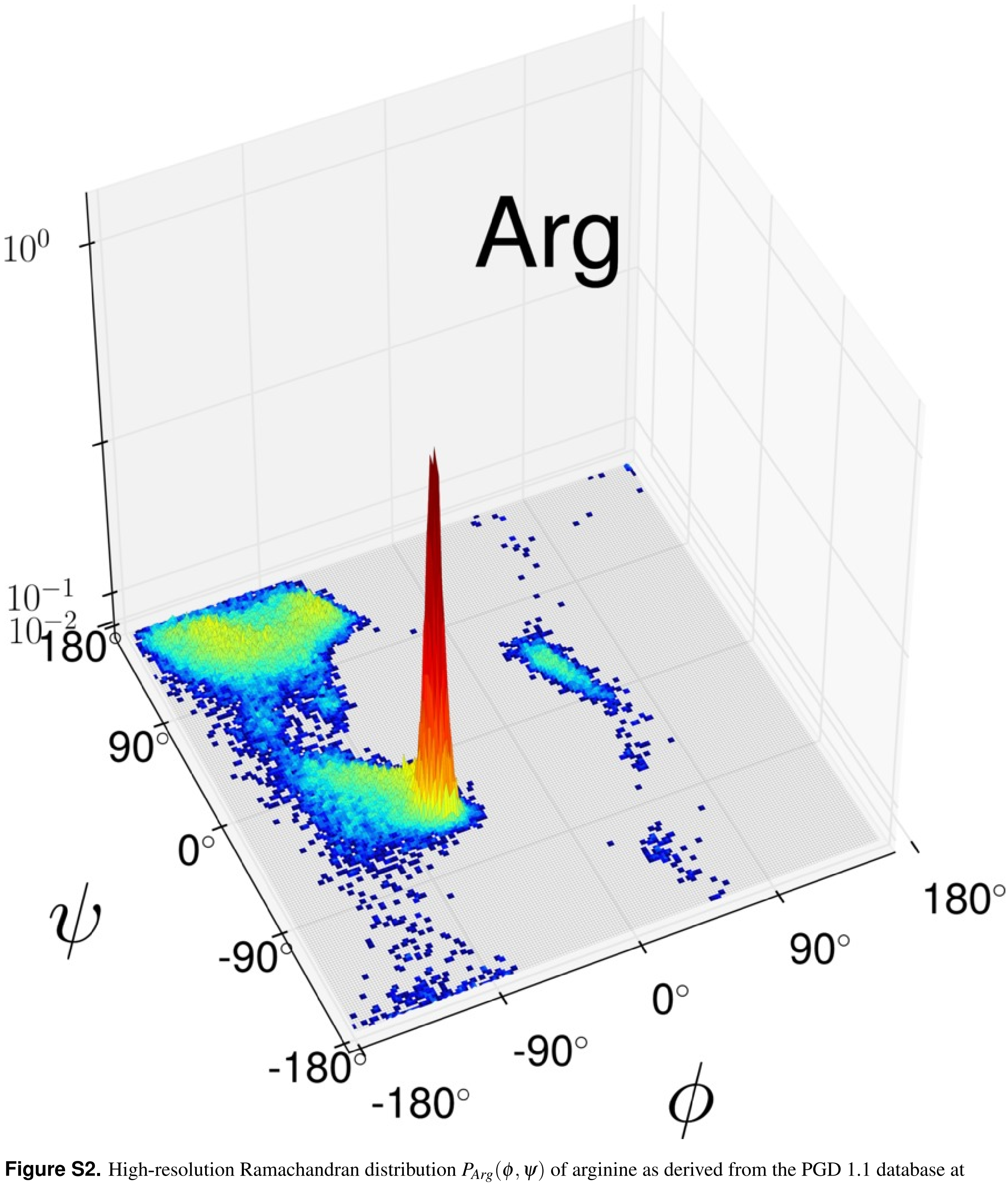

Figure S2. High-resolution Ramachandran distribution $P_{Arg}(\phi,\psi)$ of arginine as derived from the PGD 1.1 database at $1.895^\circ \times 1.895^\circ$ bin size (logartihmic scale).

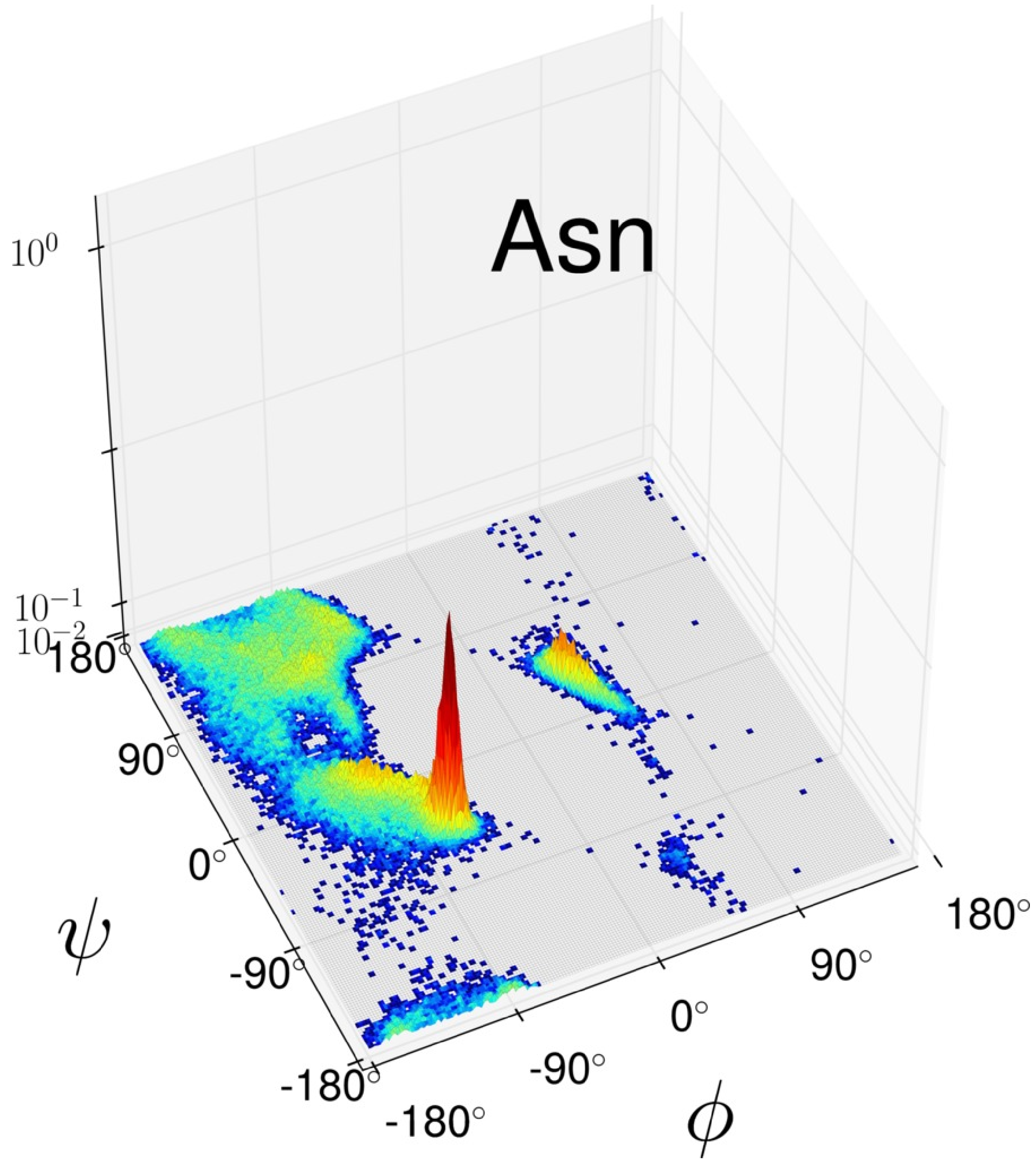

Figure S3. High-resolution Ramachandran distribution $P_{Asn}(\phi, \psi)$ of asparagine as derived from the PGD 1.1 database at $1.895^\circ \times 1.895^\circ$ bin size (logartihmic scale).

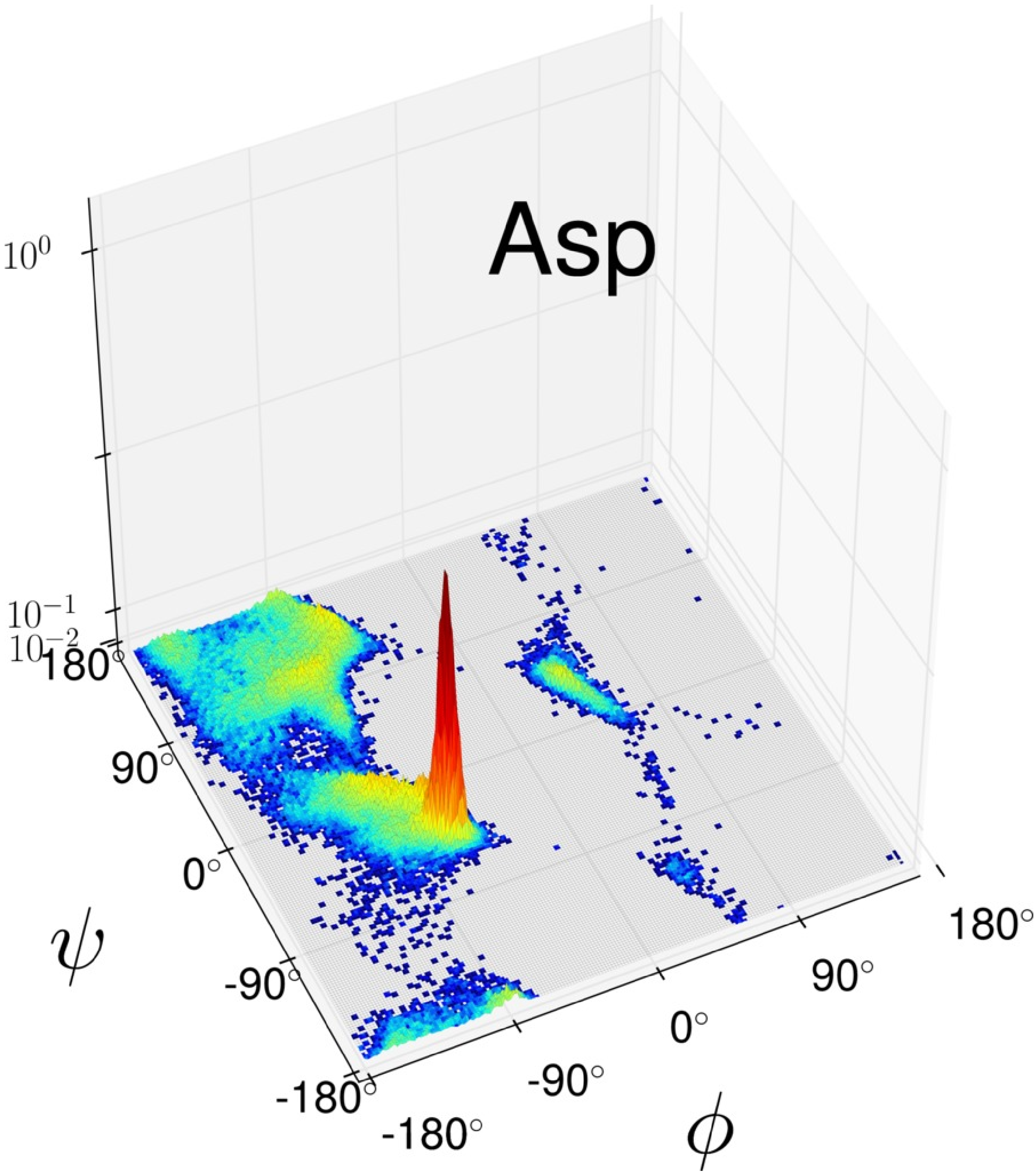

Figure S4. High-resolution Ramachandran distribution $P_{Asp}(\phi, \psi)$ of aspartic acid as derived from the PGD 1.1 database at $1.895^\circ \times 1.895^\circ$ bin size (logartihmic scale).

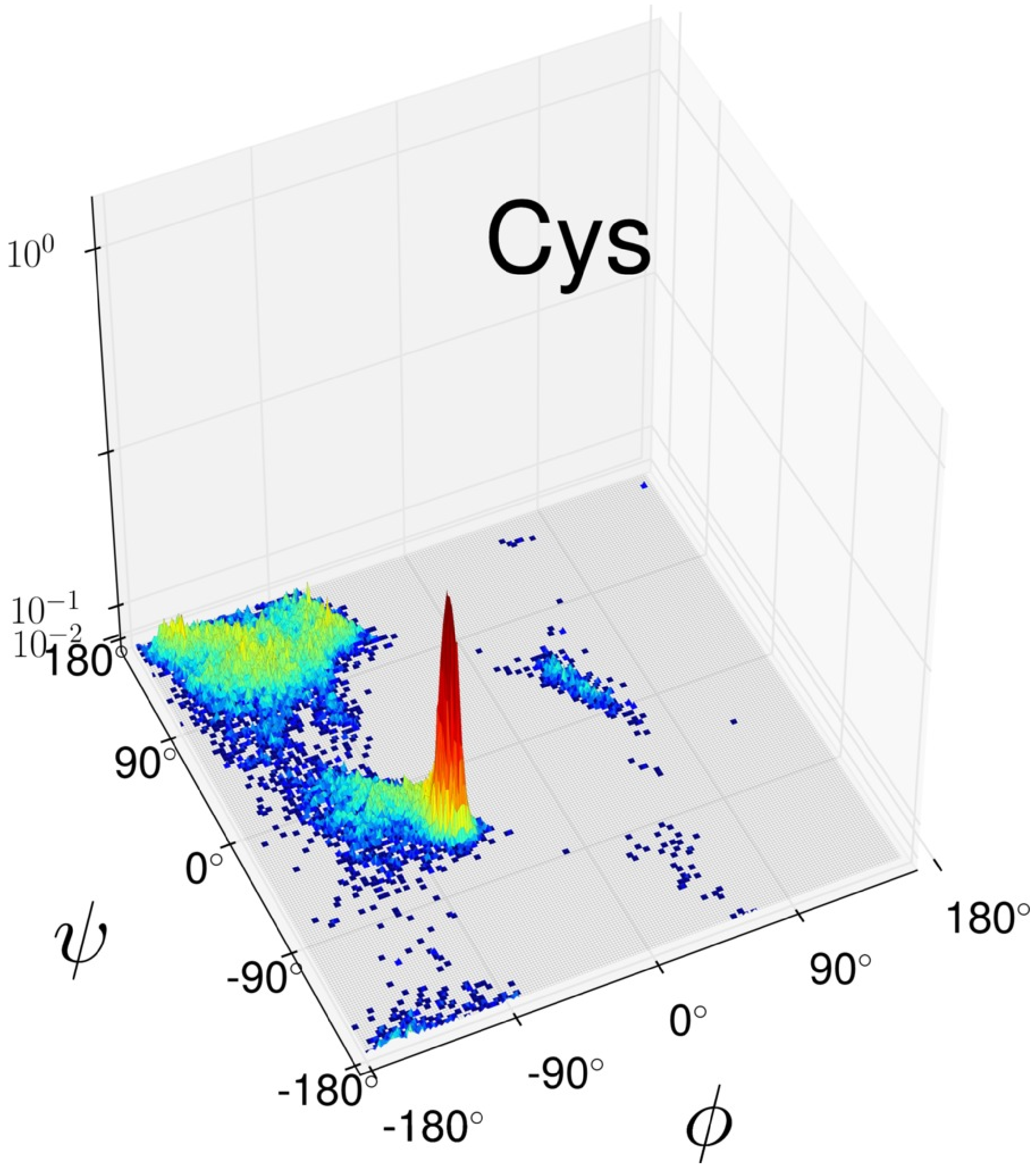

Figure S5. High-resolution Ramachandran distribution $P_{Cys}(\phi,\psi)$ of cysteine as derived from the PGD 1.1 database at $1.895^\circ \times 1.895^\circ$ bin size (logartihmic scale).

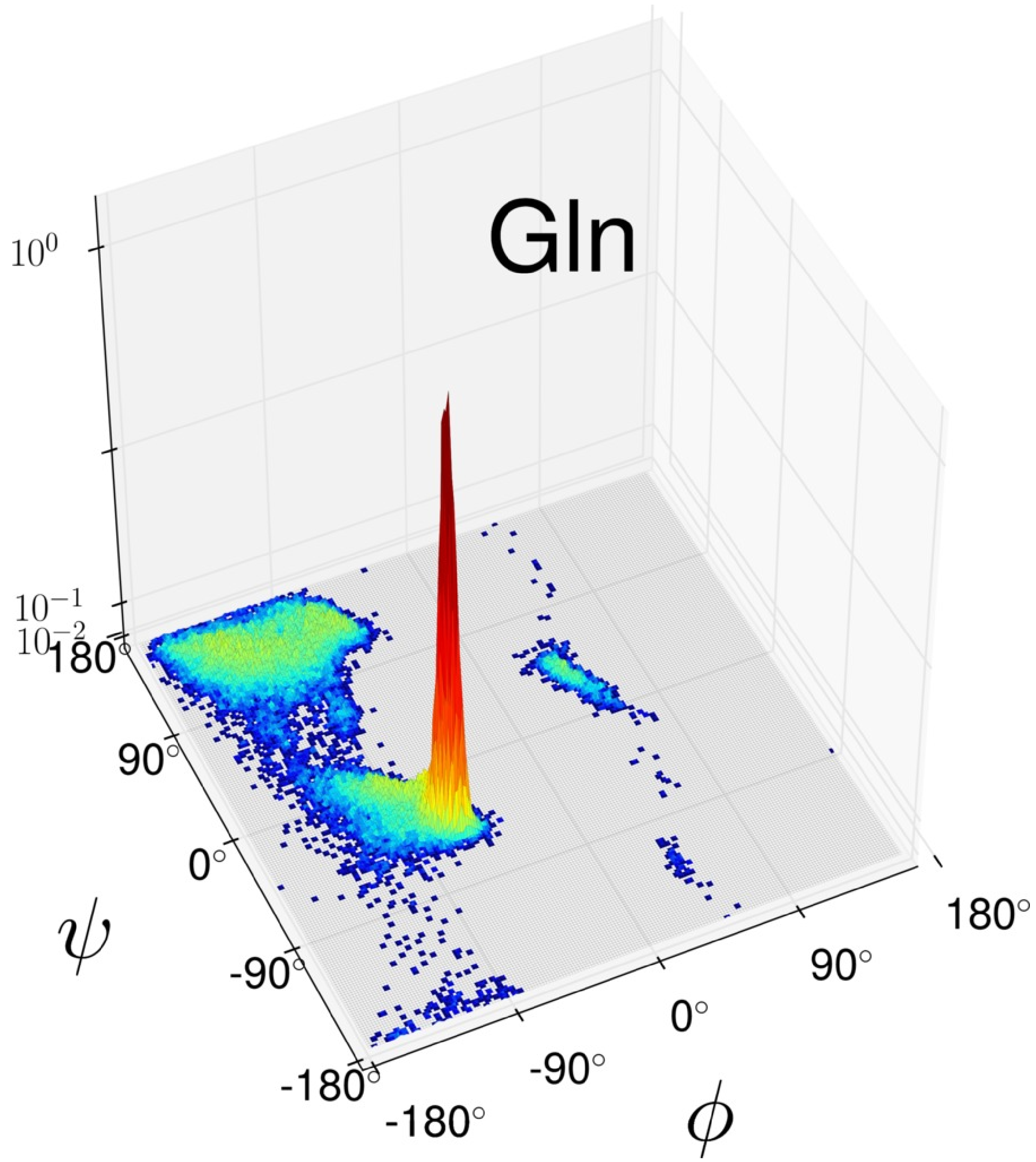

Figure S6. High-resolution Ramachandran distribution $P_{Gln}(\phi,\psi)$ of glutamine as derived from the PGD 1.1 database at $1.895^\circ \times 1.895^\circ$ bin size (logartihmic scale).

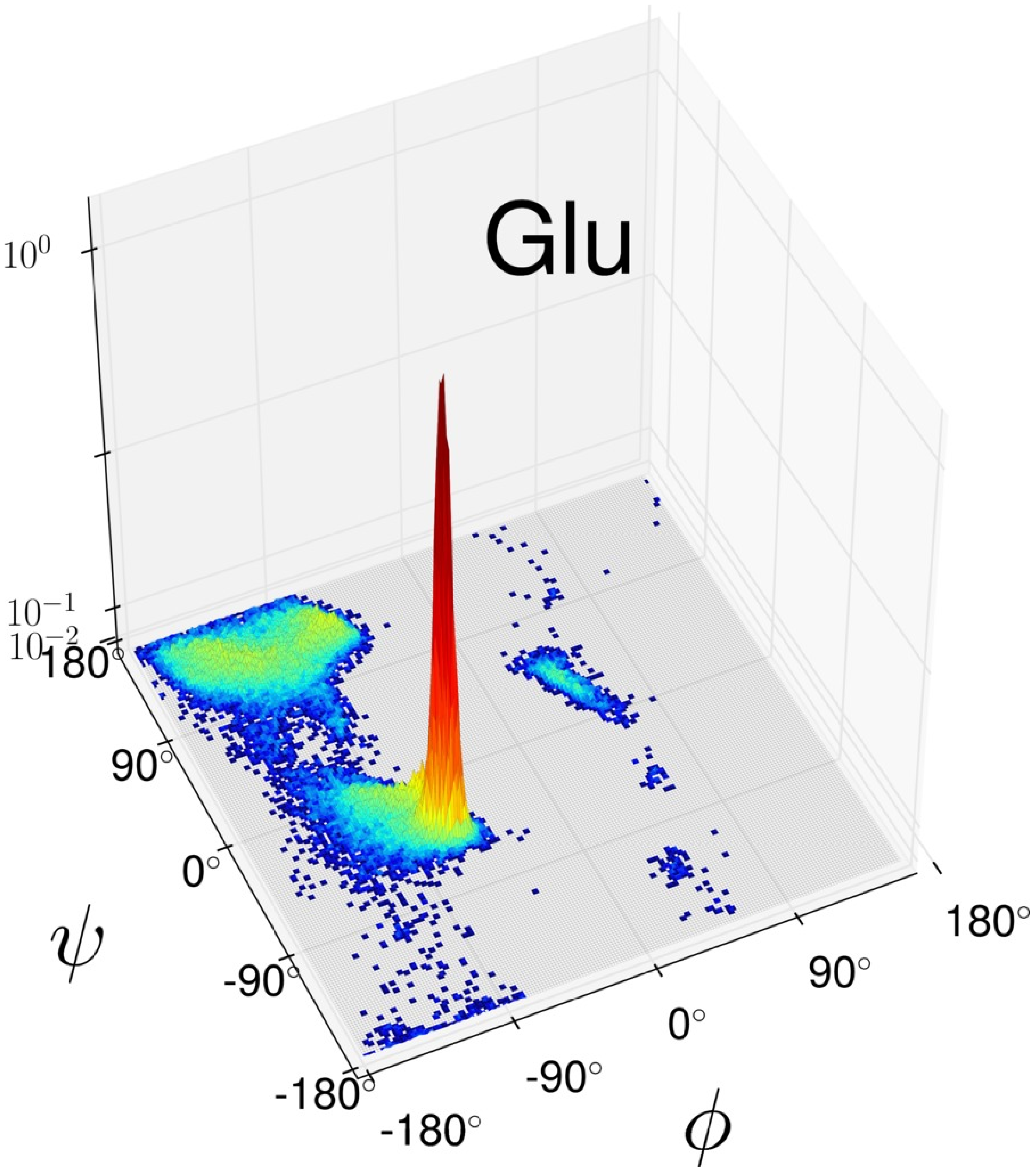

Figure S7. High-resolution Ramachandran distribution $P_{Glu}(\phi,\psi)$ of glutamic acid as derived from the PGD 1.1 database at $1.895^\circ \times 1.895^\circ$ bin size (logartihmic scale).

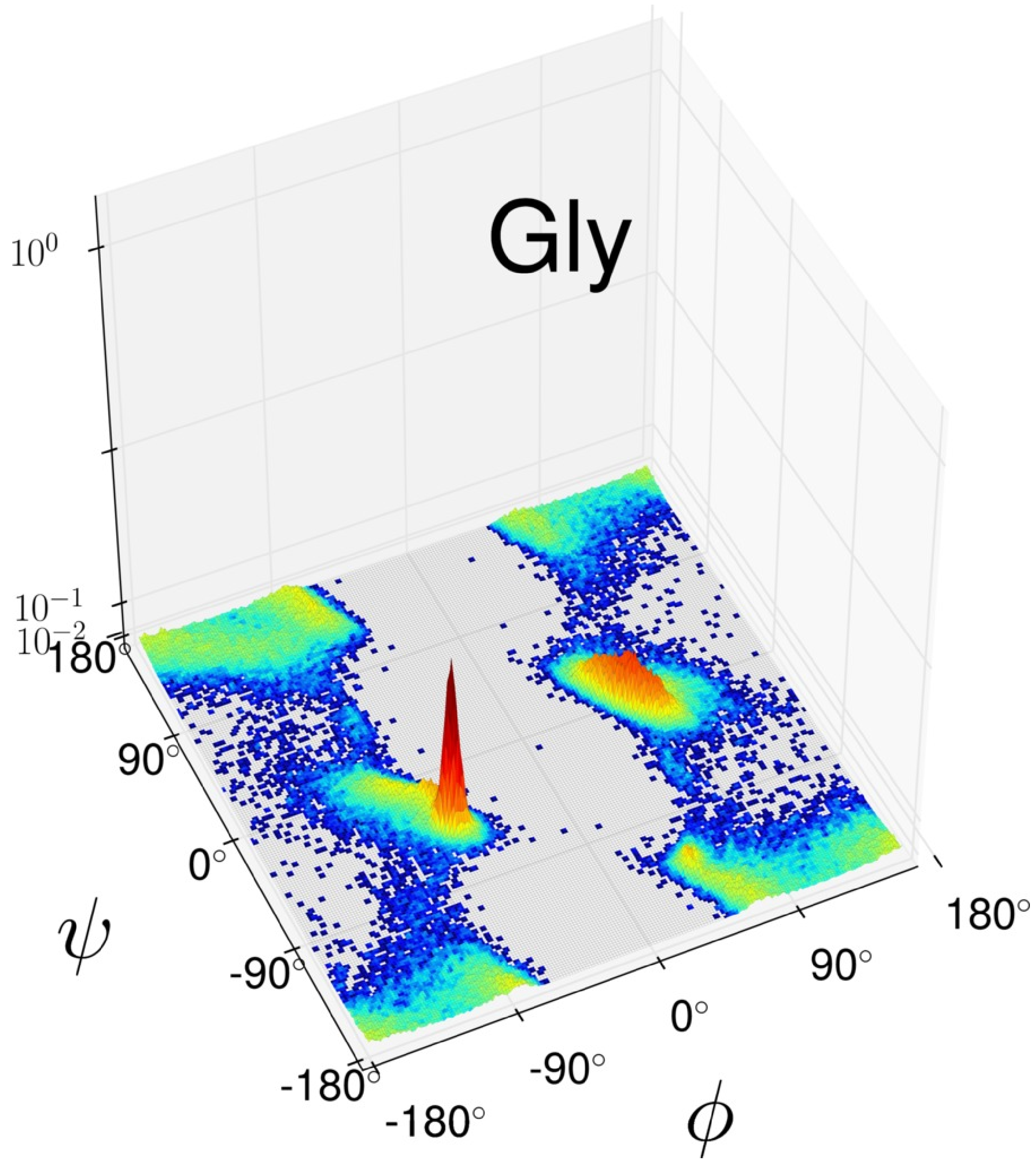

Figure S8. High-resolution Ramachandran distribution $P_{Gly}(\phi, \psi)$ of glycine as derived from the PGD 1.1 database at $1.895^\circ \times 1.895^\circ$ bin size (logartihmic scale).

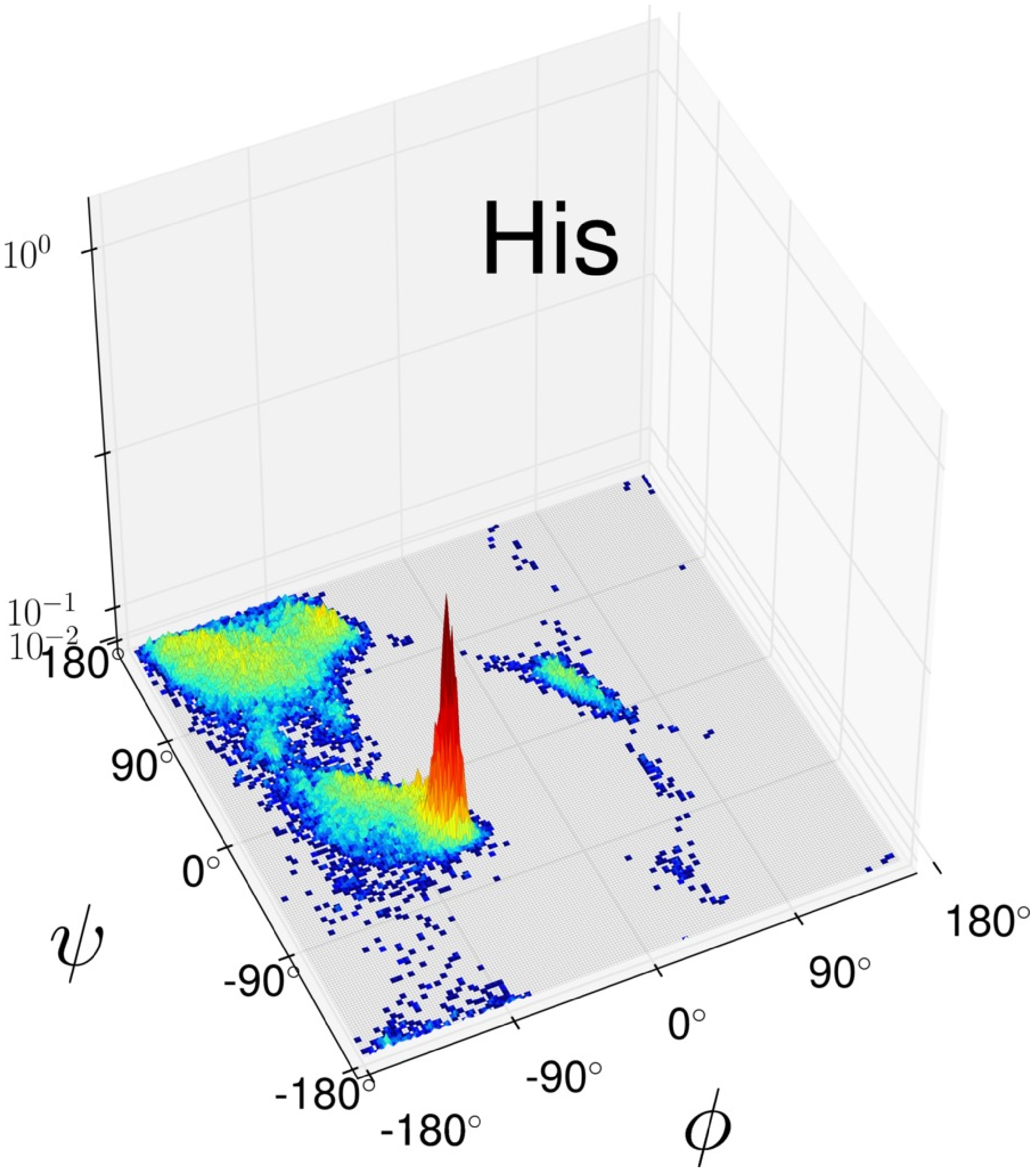

Figure S9. High-resolution Ramachandran distribution $P_{His}(\phi,\psi)$ of histidine as derived from the PGD 1.1 database at $1.895^\circ \times 1.895^\circ$ bin size (logartihmic scale).

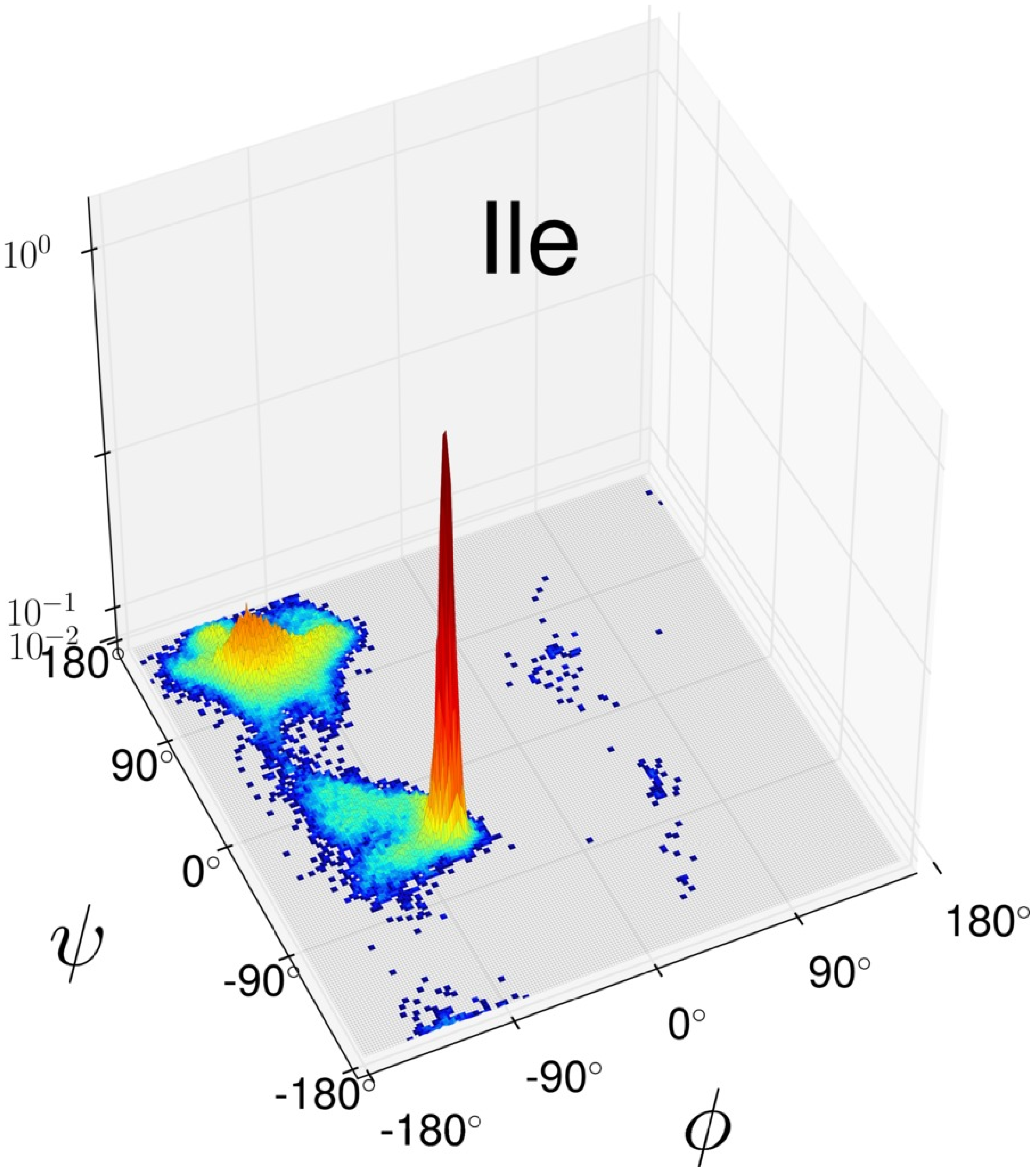

Figure S10. High-resolution Ramachandran distribution $P_{Ile}(\phi, \psi)$ of isoleucine as derived from the PGD 1.1 database at $1.895^\circ \times 1.895^\circ$ bin size (logartihmic scale).

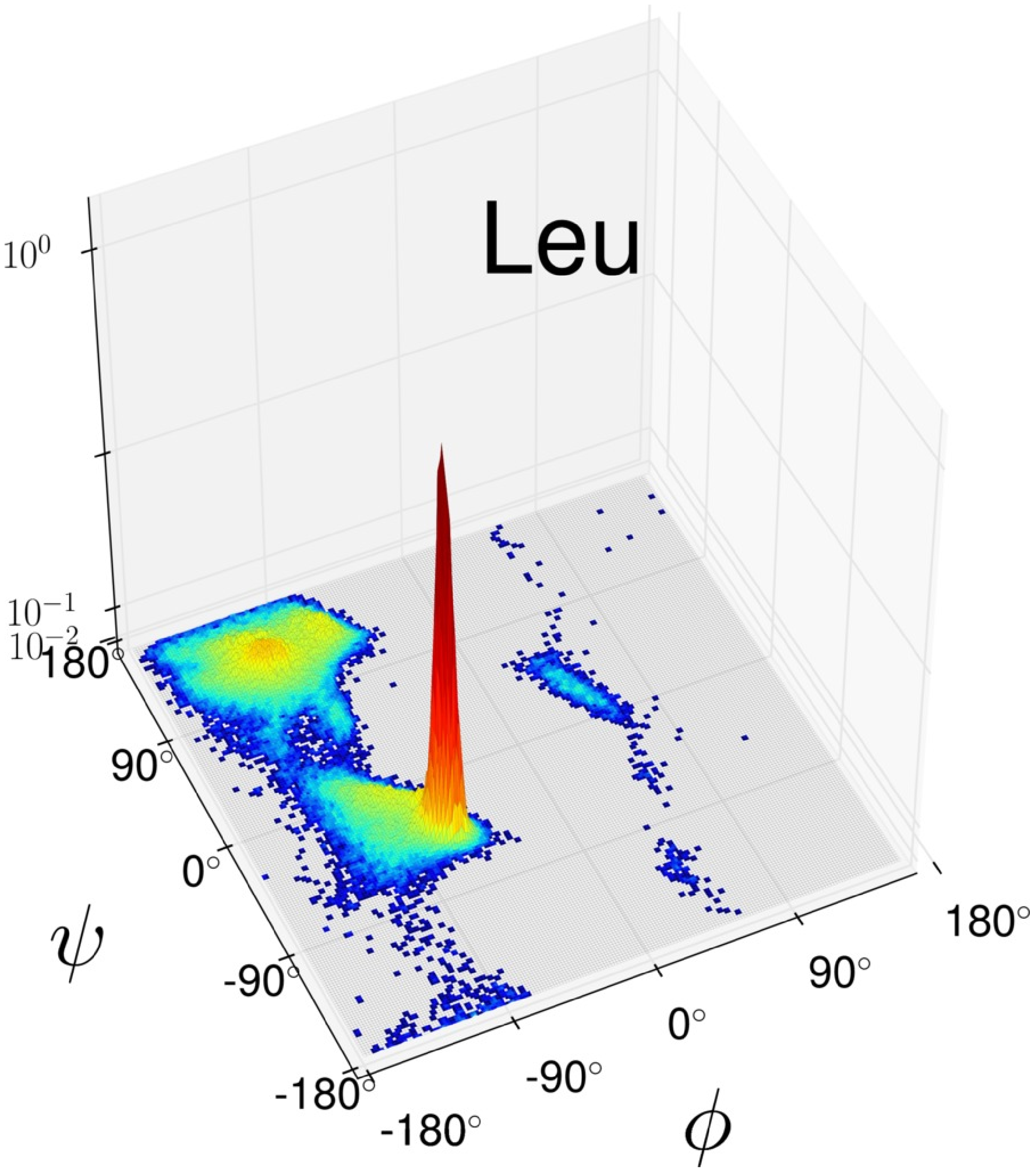

Figure S11. High-resolution Ramachandran distribution $P_{LeuX}(\phi,\psi)$ of leucine as derived from the PGD 1.1 database at $1.895^\circ \times 1.895^\circ$ bin size (logartihmic scale).

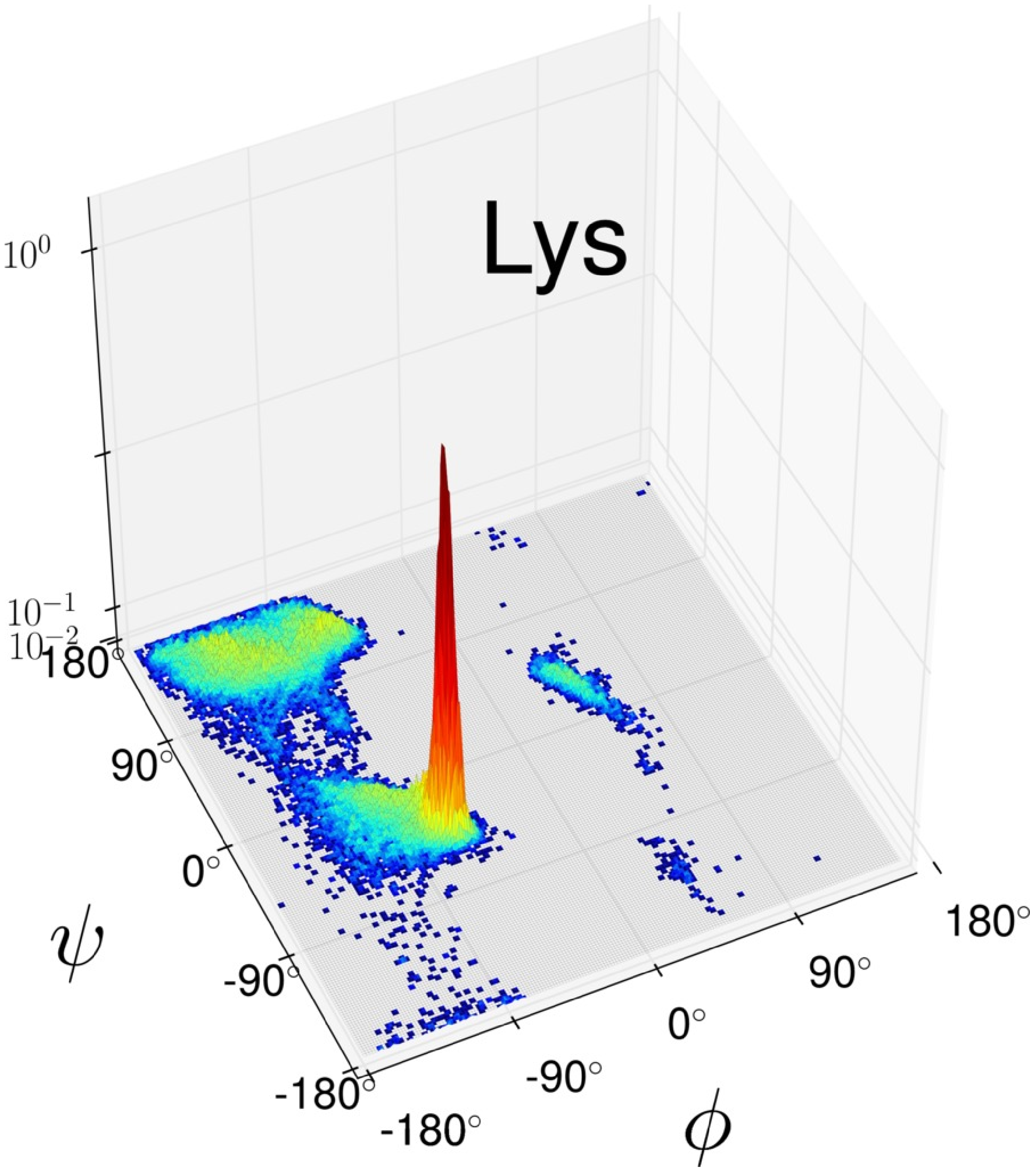

Figure S12. High-resolution Ramachandran distribution $P_{Lys}(\phi,\psi)$ of lysine as derived from the PGD 1.1 database at $1.895^\circ \times 1.895^\circ$ bin size (logartihmic scale).

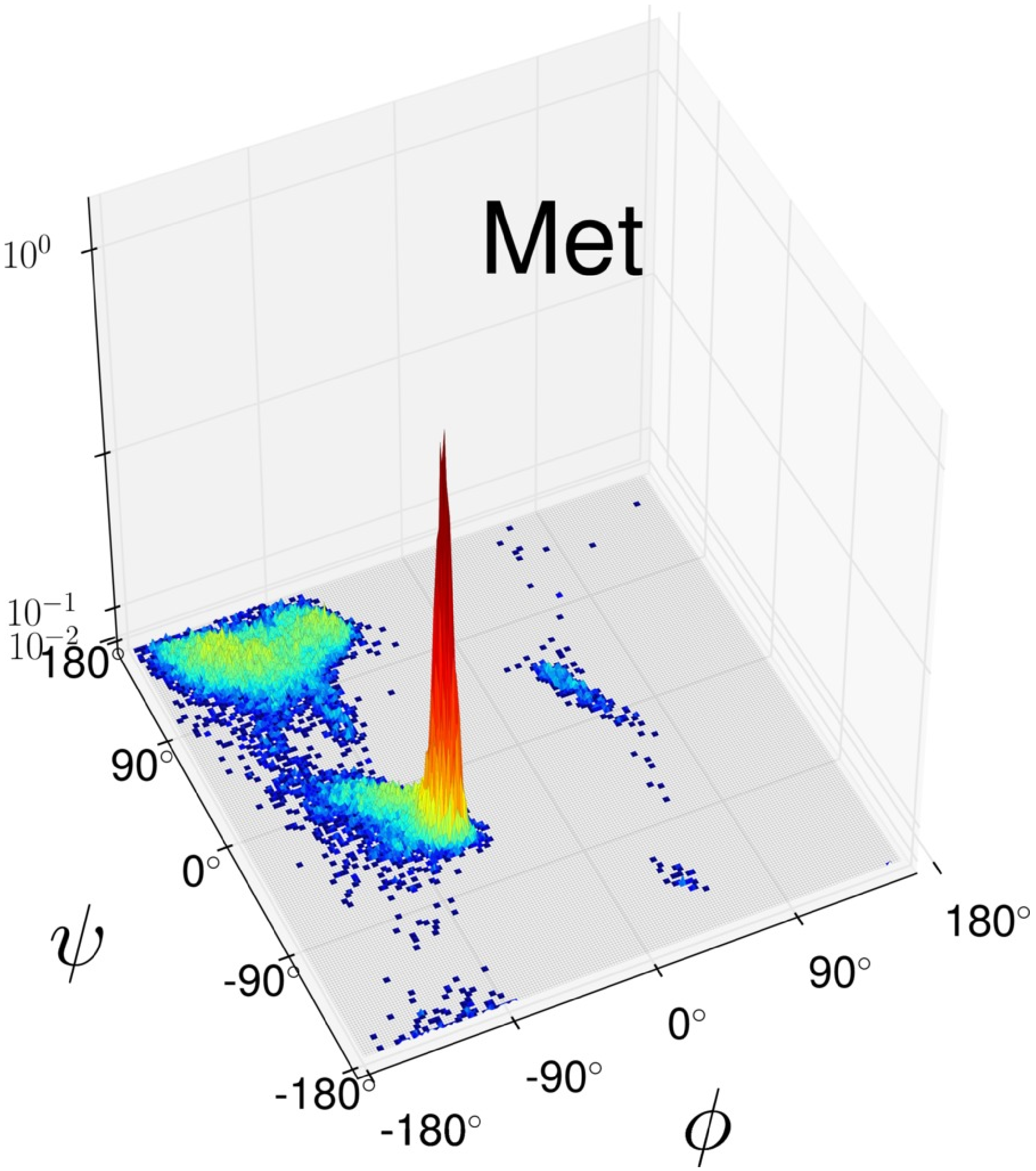

Figure S13. High-resolution Ramachandran distribution $P_{Met}(\phi, \psi)$ of methionine as derived from the PGD 1.1 database at $1.895^\circ \times 1.895^\circ$ bin size (logartihmic scale).

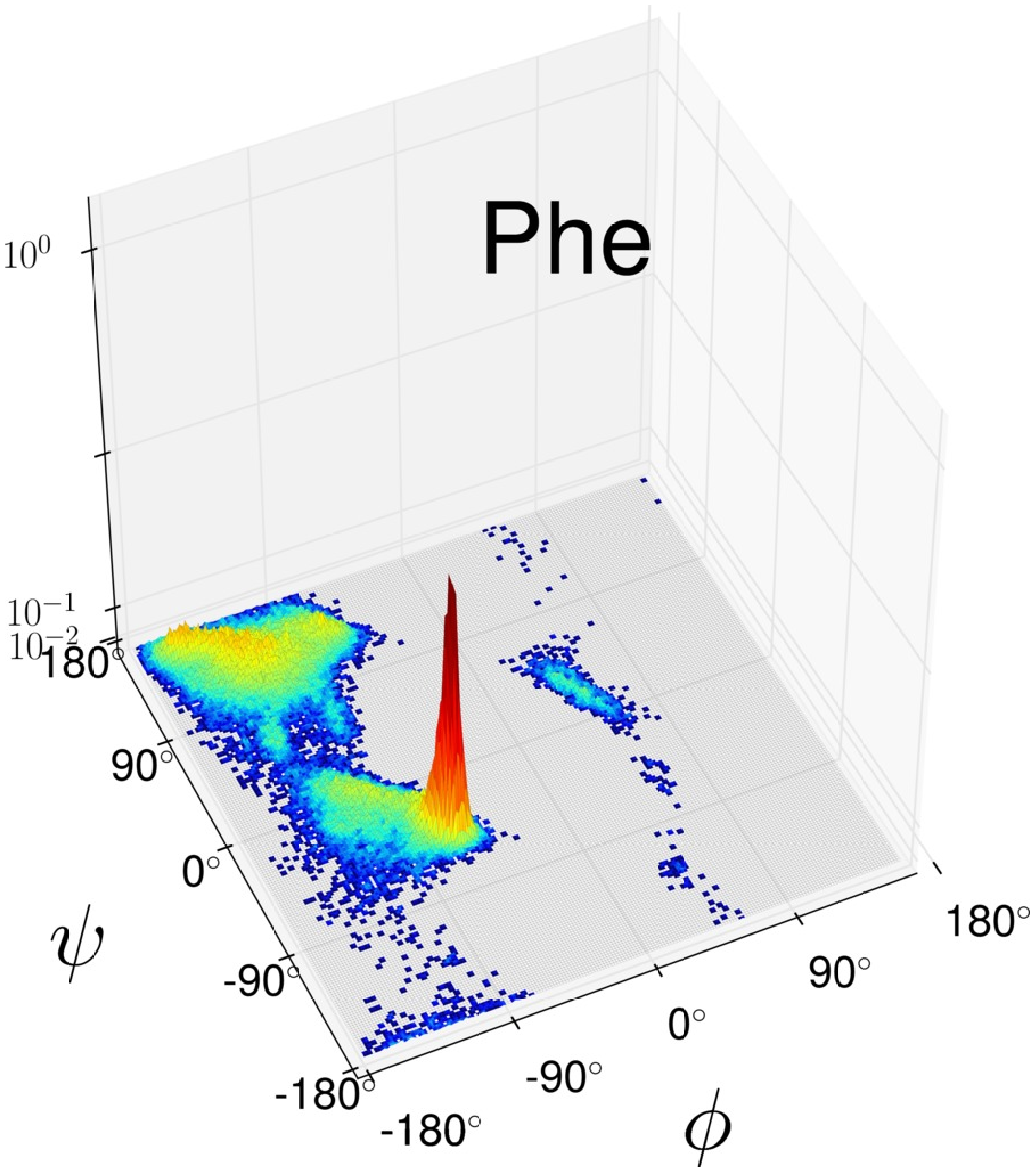

Figure S14. High-resolution Ramachandran distribution $P_{Phe}(\phi, \psi)$ of phenilalanine as derived from the PGD 1.1 database at $1.895^{\circ} \times 1.895^{\circ}$ bin size (logartihmic scale).

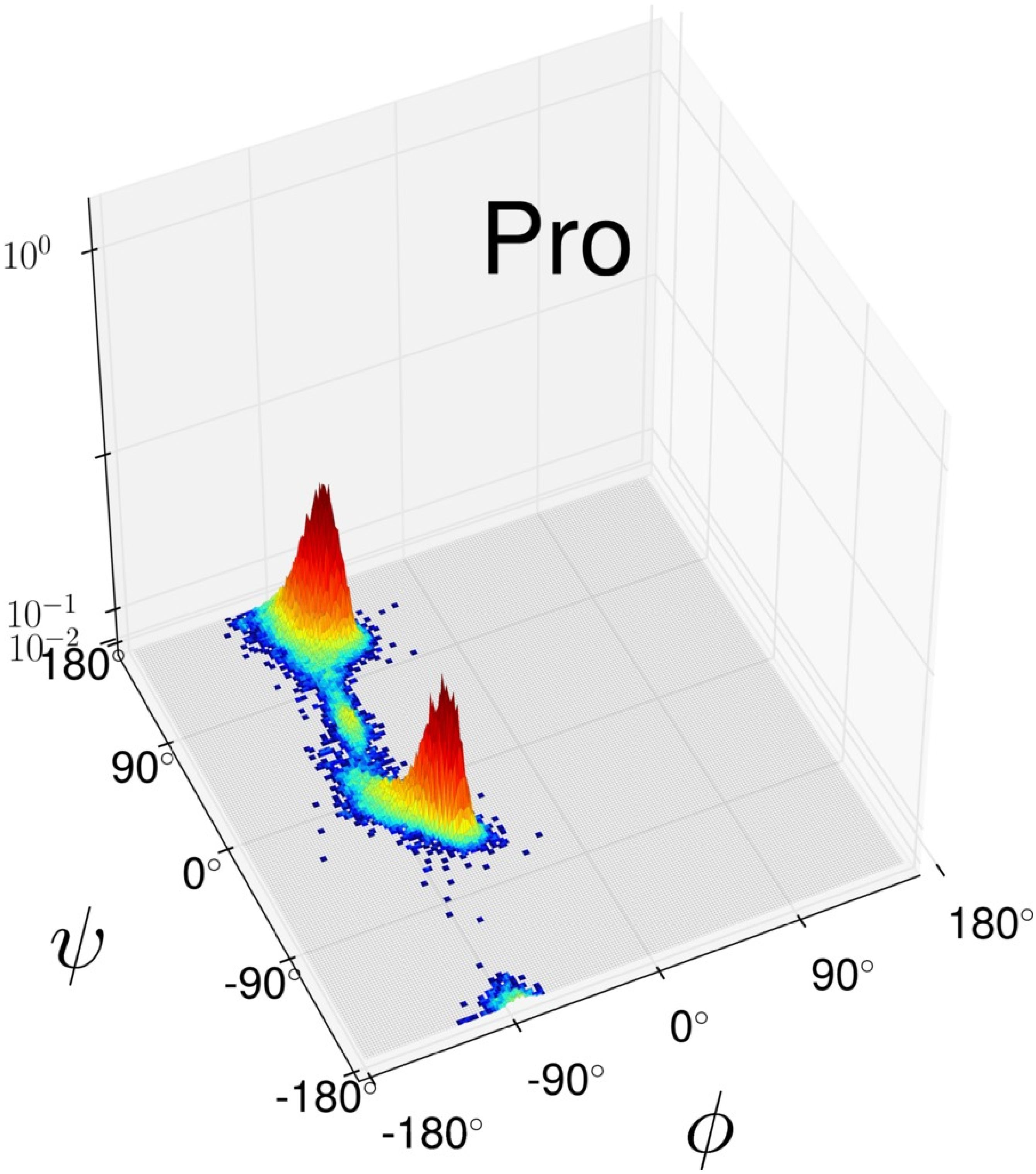

Figure S15. High-resolution Ramachandran distribution $P_{Pro}(\phi, \psi)$ of proline as derived from the PGD 1.1 database at $1.895^\circ \times 1.895^\circ$ bin size (logartihmic scale).

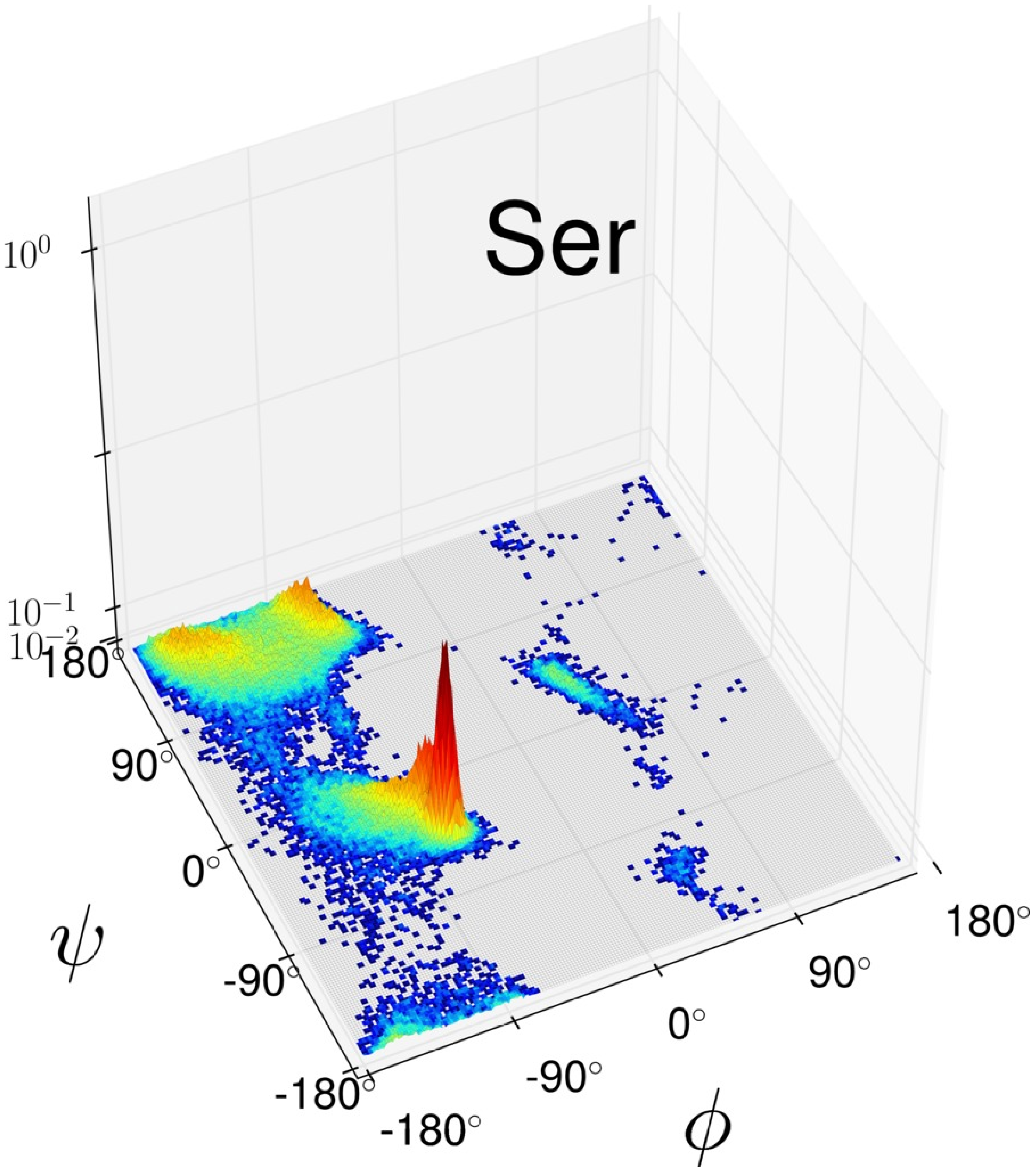

Figure S16. High-resolution Ramachandran distribution $P_{Ser}(\phi, \psi)$ of serine as derived from the PGD 1.1 database at $1.895° \times 1.895°$ bin size (logartihmic scale).

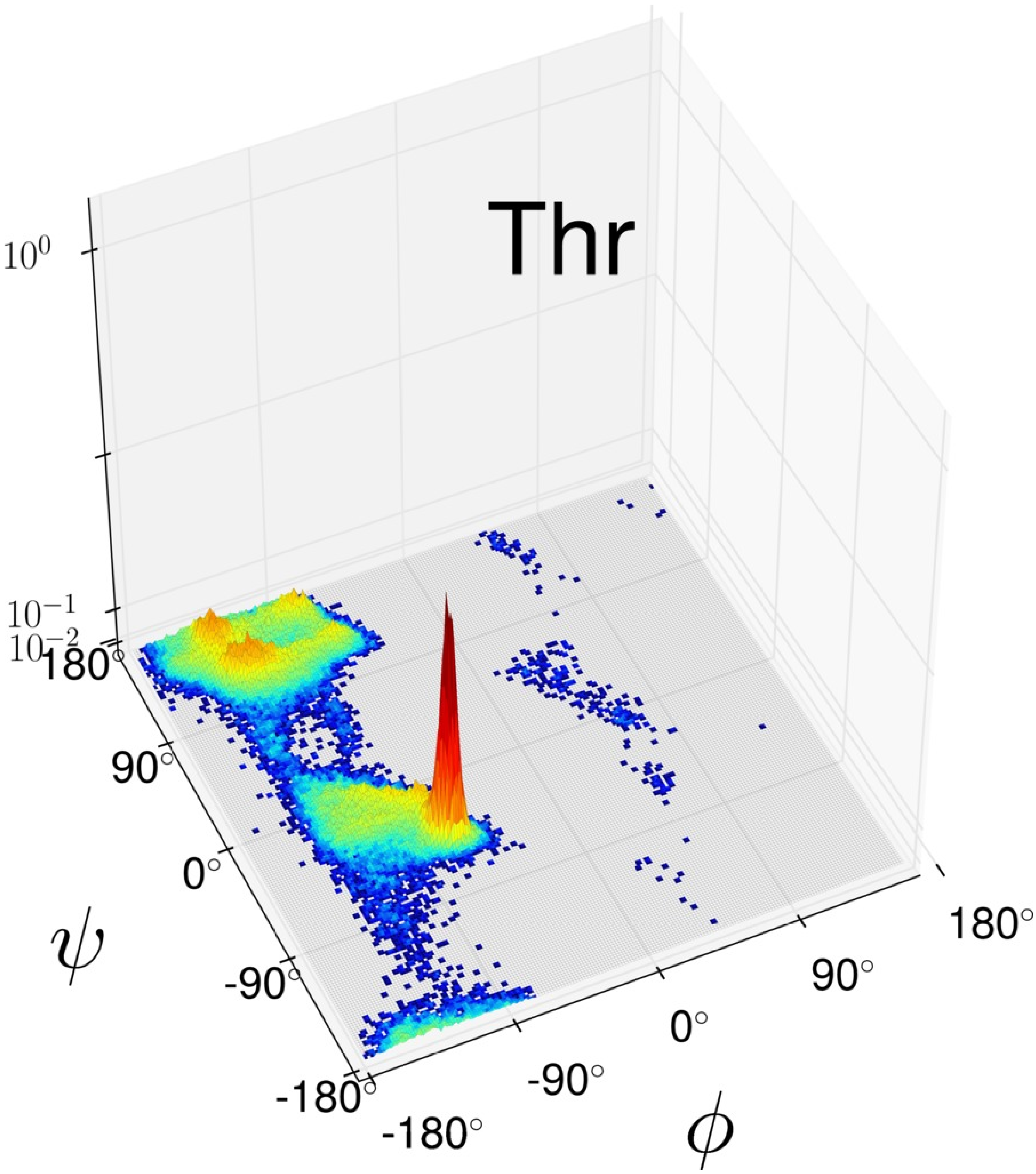

Figure S17. High-resolution Ramachandran distribution $P_{Thr}(\phi,\psi)$ of threonine as derived from the PGD 1.1 database at $1.895^\circ \times 1.895^\circ$ bin size (logartihmic scale).

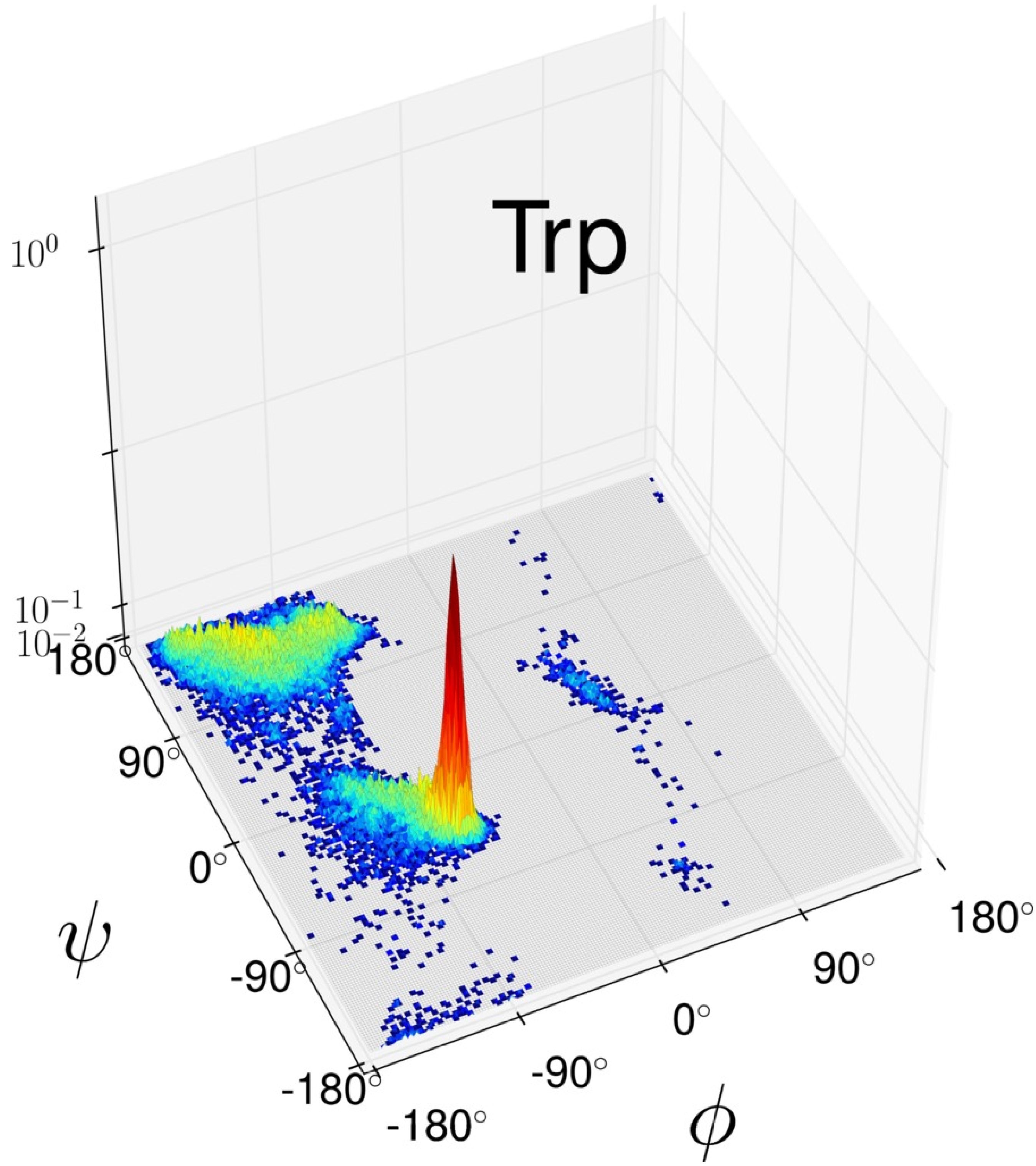

Figure S18. High-resolution Ramachandran distribution $P_{Trp}(\phi,\psi)$ of tryptophan as derived from the PGD 1.1 database at $1.895^\circ \times 1.895^\circ$ bin size (logartihmic scale).

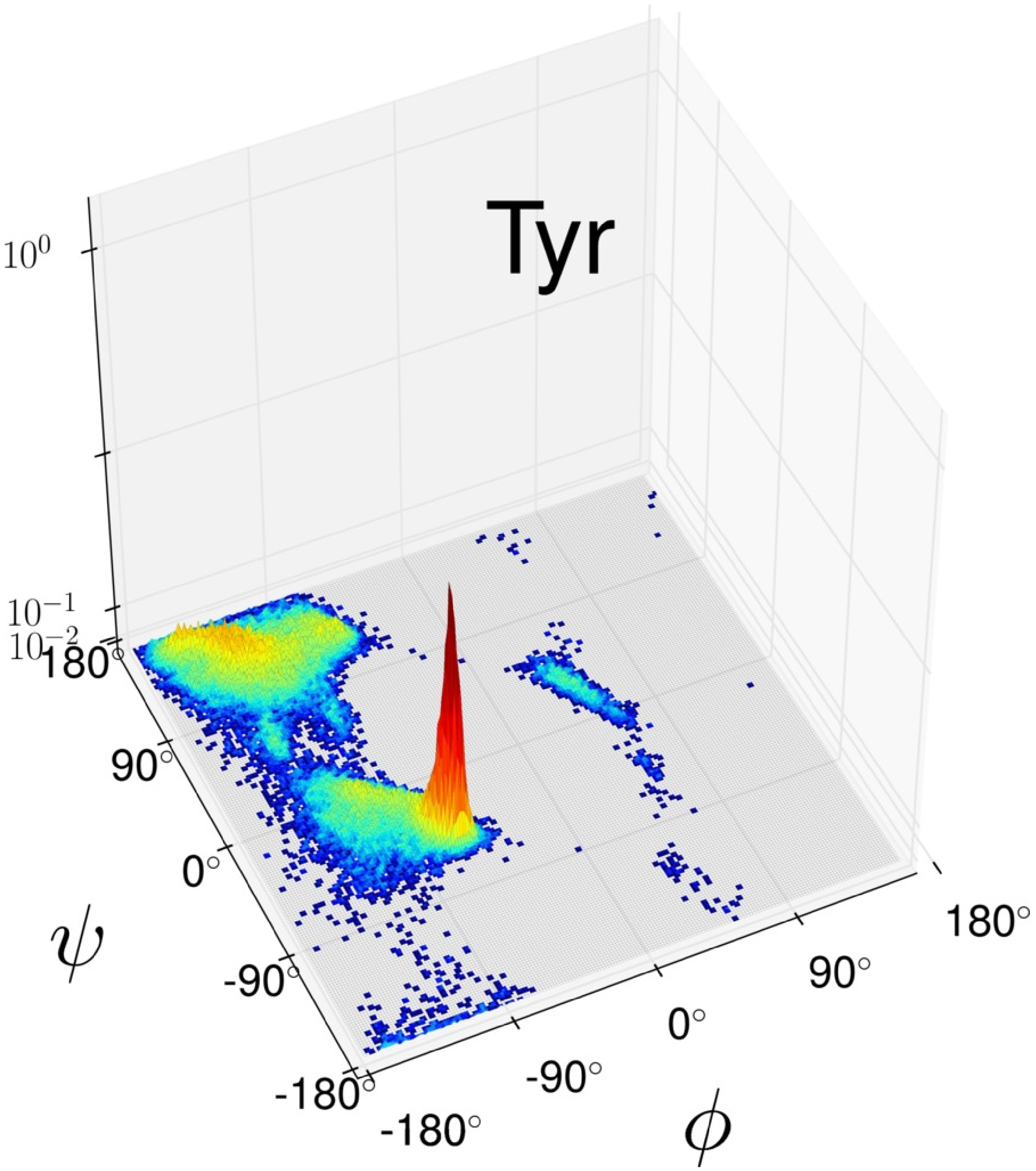

Figure S19. High-resolution Ramachandran distribution $P_{Tyr}(\phi, \psi)$ of tyrosine as derived from the PGD 1.1 database at $1.895^\circ \times 1.895^\circ$ bin size (logartihmic scale).

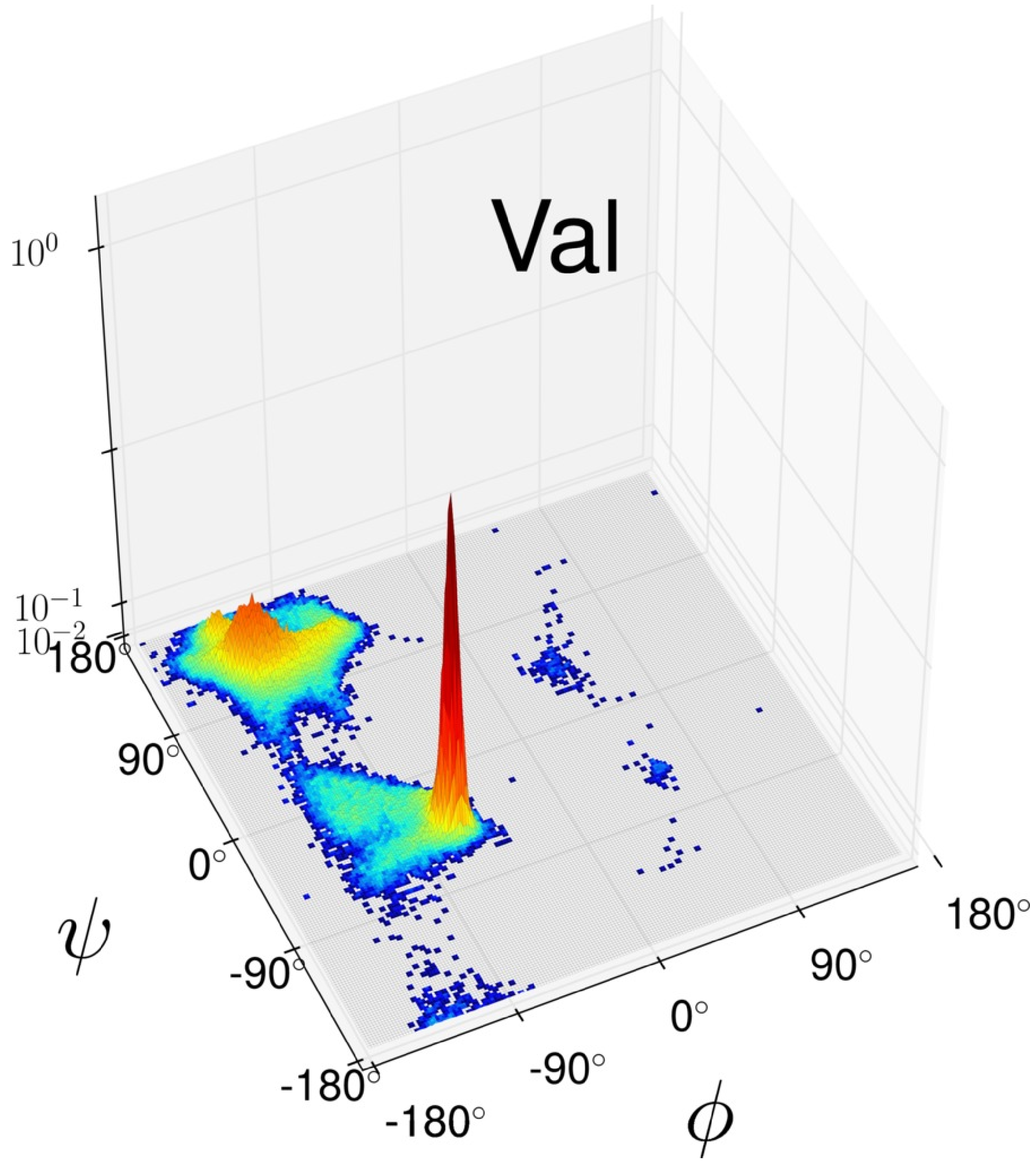

Figure S20. High-resolution Ramachandran distribution $P_{Val}(\phi, \psi)$ of valine as derived from the PGD 1.1 database at $1.895^\circ \times 1.895^\circ$ bin size (logartihmic scale).

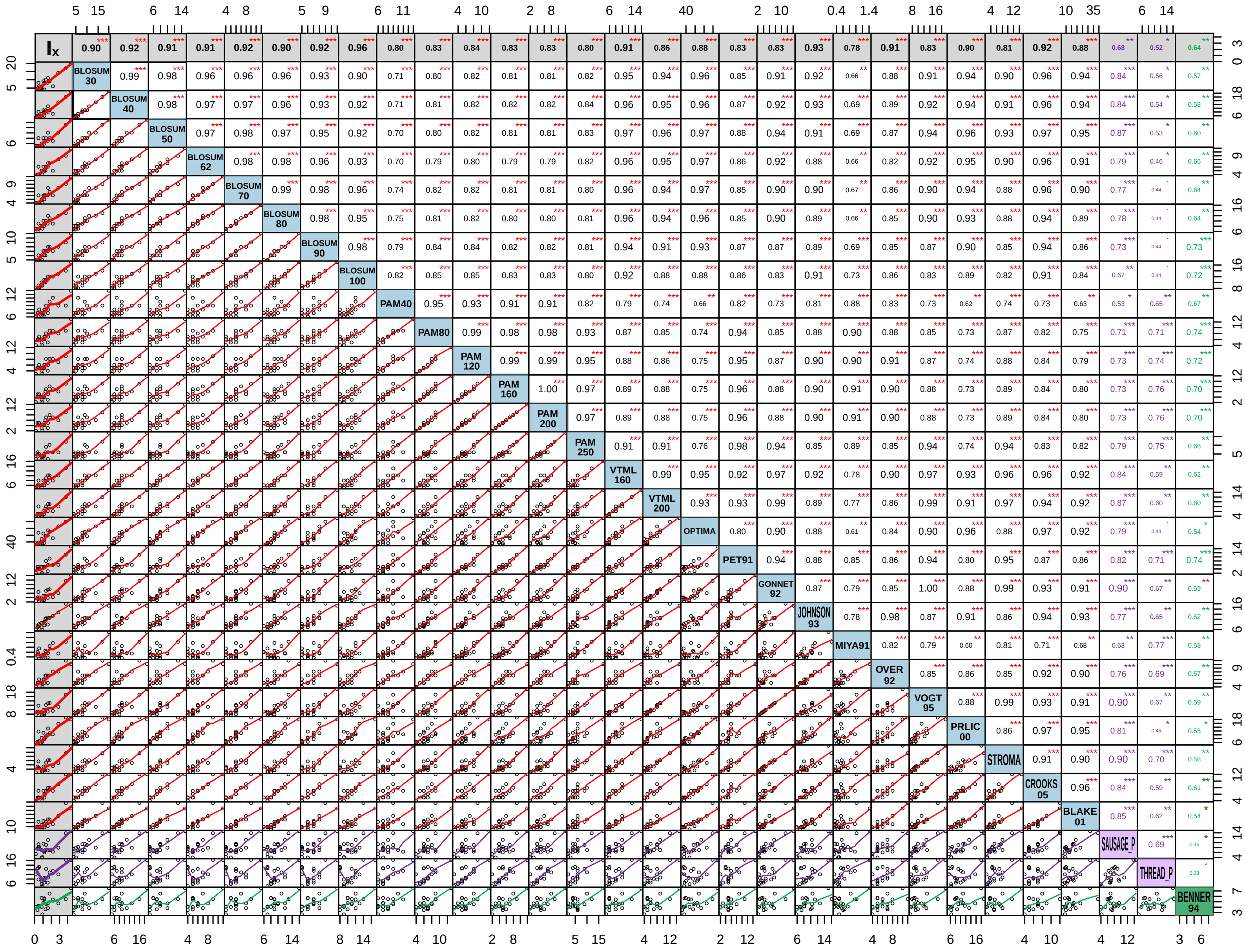

Figure S21. Correlation matrix plot with significance levels between the replacement inertia I_X and the mutability of the full set of replacement matrices used in the present study (Table S1). The lower triangular matrix is composed by the bivariate scatter plots with a fitted smooth line. The upper triangular matrix show the Pearson correlation plus significance level (as stars). Each significance level is associated to a symbol: p-values 0.001 (***), 0.01 (**), 0.05 (*). This plot was generated with the Performance Analytics package in the R program.[3] The abbreviations used in this plot are detailed in Table S1.

Table S1. Abbreviations used in the present study (left) and the corresponding description (center) of the set of substitution matrices with their respective source or AAindex code (right).

Name	Description	AAindex Entry/Source
BLOSUM30	The BLOSUM30 matrix	ftp://ftp.ncbi.nih.gov/blast/matrices/
BLOSUM40	The BLOSUM40 matrix	ftp://ftp.ncbi.nih.gov/blast/matrices/
BLOSUM50	The BLOSUM50 matrix	HENS920104
BLOSUM62	The BLOSUM62 matrix	HENS920102
BLOSUM70	The BLOSUM70 matrix	HENS920103
BLOSUM80	The BLOSUM80 matrix	ftp://ftp.ncbi.nih.gov/blast/matrices/
BLOSUM90	The BLOSUM90 matrix	ftp://ftp.ncbi.nih.gov/blast/matrices/
BLOSUM100	The BLOSUM100 matrix	ftp://ftp.ncbi.nih.gov/blast/matrices/
PAM40	The PAM40 matrix	DAYM780302
PAM80	The PAM80 matrix	ftp://ftp.ncbi.nih.gov/blast/matrices/
PAM120	The PAM120 matrix	ALTS910101
PAM160	The PAM160 matrix	ftp://ftp.ncbi.nih.gov/blast/matrices/
PAM200	The PAM200 matrix	ftp://ftp.ncbi.nih.gov/blast/matrices/
PAM250	The PAM250 matrix	DAYM780301
VTML160	The VTML160 matrix	MUET020101
VTML200	The VTML250 matrix	MUET020102
OPTIMA	The OPTIMA matrix	KANM000101
PET91	The 250 PAM PET91 matrix	JOND920103
GONNET92	The mutation matrix for initially aligning	GONG920101
JOHNSON93	Structure-based amino acid scoring table	JOHM930101
MIYA91	Base-substitution-protein-stability matrix	MIYS930101
OVER92	STR matrix from structure-based alignments	OVEJ920101
VOGT95	Amino acid exchange matrix	VOGG950101
PRLIC00	Homologous structure derived matrix	PRLA000102
STROMA	STROMA score matrix for the alignment of known distant homologs	QUIB020101
CROOKS05	Substitution matrix computed from the Dirichlet Mixture Model	CROG050101
BLAKE01	Matrix built from structural superposition data for identifying potential	BLAJ010101
SAUSAGE_P	Amino acid similarity matrix based on the SAUSAGE force field	DOSZ010101
THREAD_P	Amino acid similarity matrix based on the THREADER force field	DOSZ010103
BENNER94	Genetic code matrix	BENS940104

References

1. Berkholz, D. S., Krenesky, P. B., Davidson, J. R. & Karplus, P. A. Protein Geometry Database: a flexible engine to explore backbone conformations and their relationships to covalent geometry. *Nucleic Acids Res.* **38**, D320–D325 (2010).
2. Shimazaki, H. & Shinomoto, S. A method for selecting the bin size of a time histogram. *Neural Computation* **19**, 1503–1527 (2007).
3. Peterson, B. G. *et al.* Performanceanalytics: Econometric tools for performance and risk analysis. r package version 1.4. 3541 (2014).
